# Exploring the Underlying Molecular Mechanisms of Chaetomugilin Q in Breast Cancer Using Network Pharmacology, Molecular Docking, and Bioinformatics

**DOI:** 10.3390/molecules31203661

**Published:** 2026-10-13

**Authors:** Qiurui He, Weibing Li, Jiaxuan Xiang, Jingjing Mu, Yuying Miao, Zhirong Su, Jianchun Qin, Feng Wang

**Affiliations:** 1 Department of Life Science, Yuncheng University, Yuncheng 044000, China; 2 College of Plant Science, Jilin University, Changchun 130062, China; 3 Featured Fruit Quality Control and Application Laboratory, Department of Food and Health, Yuncheng University, Yuncheng 044000, China

**Keywords:** chaetomugilin Q, network pharmacology, molecular mechanisms, molecular docking, breast cancer

## Abstract

Chaetomugilin Q has demonstrated anti-tumor effects in preclinical models. However, the potential mechanisms of chaetomugilin Q in treating breast cancer remain unclear. This study used network pharmacology and molecular docking prediction to explore these mechanisms and prioritize candidate targets of chaetomugilin Q in breast cancer. PharmMapper, SuperPred, and GalaxySagittarius-AF were utilized to obtain targets associated with chaetomugilin Q, while GeneCards, CTD, DrugBank, OMIM, and TTD were used to collect breast cancer-related targets. A total of 299 overlapping candidate targets were obtained using Venny 2.1.0. Subsequently, Gene Ontology (GO) and Kyoto Encyclopedia of Genes and Genomes (KEGG) enrichment analyses were performed using the DAVID tool. These candidate targets were input into the STRING database and Cytoscape 3.10.1 software to construct a protein–protein interaction (PPI) network and prioritize candidate core targets. Molecular docking prediction was performed using the CB-Dock2 server. The candidate core targets were further analyzed using GEPIA, HPA, cBioPortal, Kaplan–Meier Plotter, and TIMER. After GO and KEGG enrichment analyses, 1174 relevant terms and 169 signaling pathways were identified, respectively. PIK3R1, HSP90AA1, SRC, AKT1, STAT3, PIK3CD, PIK3CB, EGFR, GRB2, and ESR1 were prioritized as candidate core targets. Molecular docking results showed that the predicted docking scores of chaetomugilin Q to the candidate core targets were lower than −5 kcal/mol. Bioinformatics analyses based on independent public databases indicated that these candidate core targets exhibited differential expression at the mRNA and protein levels and were associated with patient prognosis, mutation status, and immune cell infiltration, providing clinical and biological context. This study provides a computational prediction of the potential mechanisms of chaetomugilin Q against breast cancer and a theoretical basis for subsequent experimental validation.

## 1. Introduction

Breast cancer is a malignant tumor characterized by unique epidemiological characteristics and marked heterogeneity [1]. Female gender is the greatest risk factor for breast cancer, with women accounting for approximately 99% of all cases and men for only 0.5% to 1% [2,3]. According to the report of global cancer statistics 2022 and the global burden of disease study 2023, female breast cancer is the first or second most frequently diagnosed cancer and the fourth leading cause of cancer-associated mortality worldwide [4,5,6]. In clinical practice, female breast cancer can be classified into four distinct molecular subtypes, namely luminal A, luminal B, HER2-positive, and triple-negative breast cancer (TNBC) [7]. TNBC is characterized by the lack of progesterone receptor (PR), estrogen receptor (ER), and human epidermal growth factor receptor-2 (HER2) expression, resulting in limited treatment options and poor prognosis [8,9,10]. Therefore, TNBC is the most lethal subtype of breast cancer, accounting for 10–15% of all newly diagnosed breast cancer cases [11,12]. In the United States, the 5-year relative survival rate for female breast cancer during the 2014–2020 period was 91%, indicating a generally favorable prognosis [13]. However, the survival rate for distant metastatic breast cancer remarkably declined to 32% [14,15]. Thus, development of effective agents and novel therapeutic targets for treating breast cancer is urgently needed.

Natural products, their derivatives and structural analogues are very important sources for the discovery and development of new drugs, especially in cancer therapeutic areas [16,17,18]. These bioactive compounds derived from natural products may exhibit multifaceted advantages in the prevention, treatment, and effective management of breast cancer [19,20]. Moreover, the application of natural products as therapeutic agents against TNBC has been extensively investigated [21]. Azaphilones are a class of fungal secondary metabolites with remarkable biological activities, including cytotoxic, antioxidant, antimicrobial, antiviral, antifungal, and anti-inflammatory activities [22,23]. Chaetomugilin Q, an azaphilone compound produced by the endophytic fungus *Chaetomium globosum* TY1 isolated from *Ginkgo biloba*, has allelopathic, cytotoxic, antimalarial, anti-SARS-CoV-2, and anti-tumor activities [24,25,26,27]. Regarding its anticancer activity, previous studies have mainly evaluated chaetomugilin Q using in vitro cytotoxicity assays, in which it showed weak-to-moderate inhibitory effects against murine leukemia L1210 and P388 cells, human small-cell lung cancer NCI-H187 cells, and human promyelocytic leukemia HL-60 cells [26,27]. These findings suggest that chaetomugilin Q has broad cytotoxic potential, but the evidence remains preliminary and is largely restricted to hematological and lung cancer cell lines. However, very little is known about the efficacy of chaetomugilin Q against breast cancer, and its underlying molecular mechanisms remain unclear. Therefore, the efficacy and mechanisms of chaetomugilin Q against breast cancer remain to be explored.

**Figure 1:**
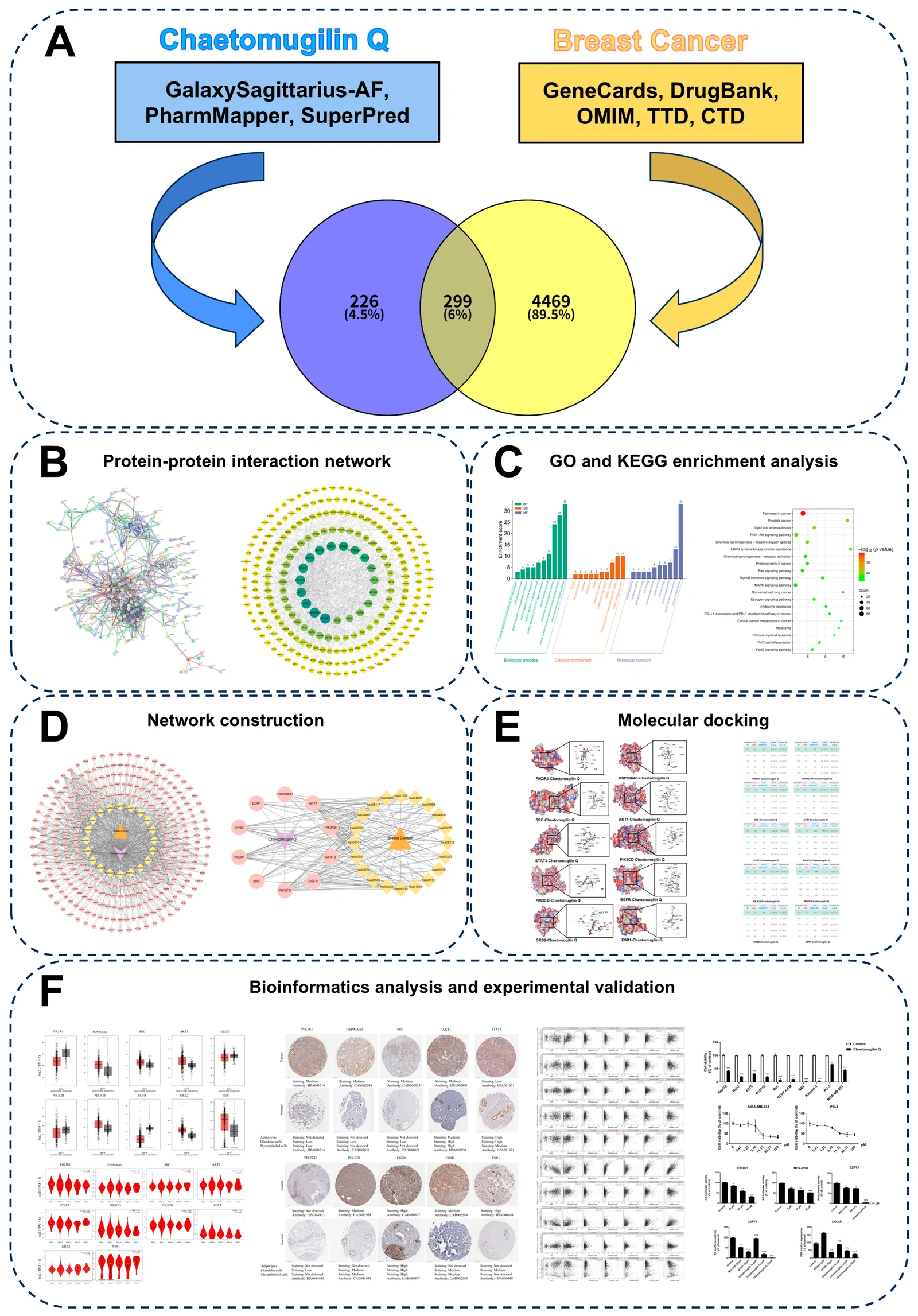
The flowchart of the study. Prediction of candidate targets (**A**). Chaetomugilin Q-related targets were retrieved from GalaxySagittarius-AF, PharmMapper, and SuperPred, while breast cancer-related targets were collected from GeneCards, DrugBank, OMIM, TTD, and CTD. The Venn diagram shows 299 overlapping candidate targets (6%). The blue and orange elements indicate chaetomugilin Q and breast cancer, respectively. Arrows indicate the workflow direction. PPI network of candidate targets (**B**). The left panel shows the global PPI network, and the right panel presents the same network in a concentric layout. Nodes represent candidate targets, and edges indicate their interactions. The inner green nodes in the right panel represent the candidate core targets, while the outer yellow nodes represent peripheral targets. GO and KEGG enrichment analysis of candidate targets (**C**). The left panel shows a bar plot of the GO enrichment analysis, categorized into Biological Process (BP, green), Cellular Component (CC, orange), and Molecular Function (MF, purple). The y-axis represents the enrichment score, and the numbers on top of the bars indicate the fold enrichment. The right panel shows a bubble plot of the KEGG pathway enrichment analysis. The y-axis represents the enriched pathways, and the x-axis represents the fold enrichment. The dot size indicates the number of enriched genes (count), and the color gradient from green to red represents the statistical significance (−log_10_(*p*-value)). Construction of compound–target–pathway–disease network (**D**). The left panel shows the full network. The right panel shows the simplified core network. In the network, chaetomugilin Q, candidate targets, KEGG pathways (hsa IDs), and breast cancer are represented by a purple V-shaped symbol, pink circles, yellow diamonds, and an orange triangle, respectively. Gray edges denote the interactions or associations among these nodes. Molecular docking analysis between chaetomugilin Q and candidate core targets (**E**). The left panel shows the predicted binding modes. The protein surface is colored by atom type (red, oxygen; blue, nitrogen; gray, carbon; yellow, sulfur). Chaetomugilin Q is depicted as a gray stick model, with its chlorine atom highlighted as a green sphere and its oxygen atoms highlighted as red spheres. Amino acid residues are shown as thin lines, and dashed lines denote non-covalent interactions (e.g., hydrogen bonds and hydrophobic contacts). The black boxes indicate the magnified views of the predicted binding pockets, with key residues labeled. The right panel shows the predicted Vina docking scores and detailed interaction parameters for each target-ligand complex. Bioinformatics analysis of candidate core targets and in vitro evaluation of chaetomugilin Q (**F**). The left panel shows box plots of the mRNA expression levels of candidate core targets in breast cancer (red, tumor tissues; gray, normal tissues) and violin plots showing the relationship between these mRNA levels and pathological stages. In the box plots, the central line denotes the median, the box encompasses the interquartile range (IQR), and the whiskers span the minimum to maximum values within 1.5 × IQR. Individual sample expression values are overlaid as dots, and a red asterisk (*) marks a statistically significant difference between the tumor and normal groups (*p* < 0.05). In the violin plots, candidate core target expression is displayed across different pathological stages of breast cancer, including Stage I–IV and Stage X (unknown). The median is indicated by a black central line, with the *F*-value and *p*-value from the ANOVA test annotated. The middle panel shows immunohistochemical images of candidate core targets in normal and breast cancer tissues from the HPA database (with staining intensity and antibody IDs labeled), alongside the associations between the expression of these targets and immune cell infiltration in breast cancer. The right panel shows the experimental assessment. Bar graphs demonstrate that chaetomugilin Q markedly suppresses the proliferation of multiple cancer cell lines, including liver cancer (HepG2 and Huh7), melanoma (A375 and B16F10), leukemia (Reh, CCRF-CEM, NB4, and Kasumi-1), prostate cancer (PC-3), and breast cancer (MDA-MB-231). Concentration-response curves illustrate the viability of MDA-MB-231 and PC-3 cells treated with increasing concentrations of chaetomugilin Q. Additionally, chaetomugilin Q significantly inhibits AR signaling in AR-positive prostate cancer cells, with the AR inhibitors EPI-001 and MDV3100 used as positive controls. Data are presented as mean ± SD or mean ± SEM (*n* = 3). * *p* < 0.05, ** *p* < 0.01, *** *p* < 0.001; ns, not significant.

Network pharmacology, initially introduced by Andrew L. Hopkins in 2007, is an innovative approach that integrates systems biology, polypharmacology, and computational techniques to overcome the limitations of conventional drug discovery [28,29,30,31]. Molecular docking is an extensively utilized method in drug discovery and development to investigate the affinities and binding modes of small molecule ligands and macromolecular receptors [32,33]. In this study, network pharmacology and molecular docking prediction were employed to computationally explore the potential mechanisms underlying the anti-breast cancer effects of chaetomugilin Q (Figure 1). These analyses suggested that chaetomugilin Q may interact with the candidate core targets, including PIK3R1, HSP90AA1, SRC, AKT1, STAT3, PIK3CD, PIK3CB, EGFR, GRB2, and ESR1, and that its predicted targets in breast cancer were primarily enriched in KEGG pathways such as Pathways in cancer, Prostate cancer, and PI3K-Akt signaling pathway. However, no direct binding or functional validation assays were performed. Although these computational predictions suggest that chaetomugilin Q may be associated with multiple candidate targets and pathways, whether it functionally modulates them remains to be determined. Further experimental studies are therefore warranted to validate these predictions.

## 2. Results

### 2.1. Prediction of Chaetomugilin Q Targets and Breast Cancer-Related Targets, Construction of PPI Networks, and Prioritization of Candidate Core Targets

**Figure 2:**
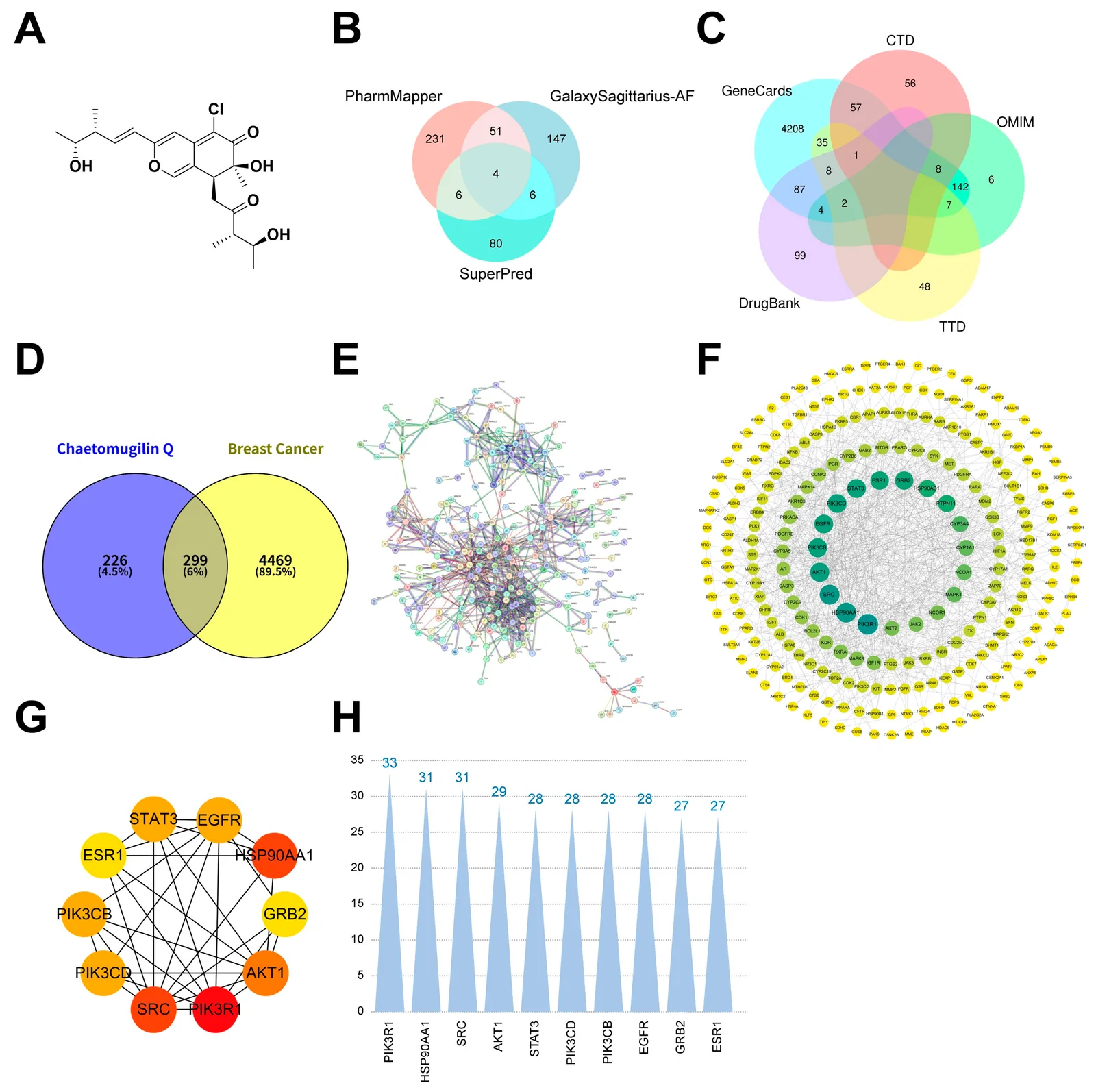
Chemical structure of chaetomugilin Q (**A**). Venn diagram of potential targets of Chaetomugilin Q predicted using PharmMapper, GalaxySagittarius-AF, and SuperPred (**B**). Different colored circles represent different target prediction databases. Numbers within each region indicate the count of predicted targets, with numbers in the non-overlapping regions representing unique targets for each database, and numbers in the overlapping regions representing shared targets among the databases. Venn diagram of breast cancer-related targets retrieved from GeneCards, CTD, OMIM, DrugBank, and TTD (**C**). Different colored circles represent different disease databases. Numbers within each region indicate the count of breast cancer-related targets, with numbers in the non-overlapping regions representing unique targets for each database, and numbers in the overlapping regions representing shared disease targets among the databases. Venn diagram of the intersecting targets between chaetomugilin Q and breast cancer (**D**). The blue and yellow circles represent the predicted targets of chaetomugilin Q and breast cancer-related targets, respectively. The overlapping region contains 299 shared targets (6%), while 226 (4.5%) and 4469 (89.5%) targets are unique to chaetomugilin Q and breast cancer, respectively. These intersecting targets were used for subsequent PPI network construction and enrichment analyses. PPI network of the intersecting targets (**E**). Nodes represent target proteins, and edges represent protein-protein interactions. Edge colors denote the types of supporting evidence (e.g., experimental, text mining, and co-expression). Colored nodes represent query proteins and first-shell interactors, while white nodes represent second-shell interactors. Additionally, filled nodes indicate that a 3D structure is known or predicted, whereas unfilled nodes lack 3D structural information. No functional clustering is indicated by node color. PPI network visualized using Cytoscape (version 3.10.1) (**F**). Nodes represent target proteins, and edges represent protein-protein interactions. The nodes are arranged in a circular layout. The color gradient (from dark green to yellow) and node size indicate the degree centrality. Nodes with higher degree centrality (inner, dark green, larger size) represent candidate core targets, while those with lower degree centrality (outer, yellow, smaller size) represent peripheral targets. Gene symbols are labeled on the nodes. PPI network of candidate core targets (**G**). Nodes represent target proteins, and edges represent protein-protein interactions. The nodes are arranged in a circular layout. The color gradient (from yellow to red) indicates the degree centrality. Nodes in red (e.g., HSP90AA1, SRC, PIK3R1) represent targets with the highest degree centrality, while nodes in yellow (e.g., ESR1, GRB2) represent targets with lower degree centrality. Degree centrality of candidate core targets (**H**). The X-axis represents the gene symbols, and the Y-axis represents the degree centrality values. The bars are arranged in descending order. The exact degree centrality value for each target is labeled above the corresponding bar.

The chemical structure of chaetomugilin Q is shown in Figure 2A. A total of 525 potential targets of chaetomugilin Q were predicted using PharmMapper, SuperPred, and GalaxySagittarius-AF (Figure 2B). A total of 4768 breast cancer-related targets were collected from five databases: GeneCards, CTD, DrugBank, OMIM, and TTD (Figure 2C). The Venn diagram shows the intersection between the obtained breast cancer-related targets and the predicted targets of chaetomugilin Q, yielding 299 overlapping candidate targets (Figure 2D). To assess potential interactions between the proteins encoded by these overlapping genes, the 299 candidate targets were subjected to PPI network analysis using the STRING database. The PPI network contained 298 nodes and 815 edges, with an average node degree of 5.47, and the PPI enrichment *p*-value was less than 1.0 × 10^−16^ (Figure 2E). The PPI data from the TSV file was imported into Cytoscape 3.10.1 software for visualization. The “Analyze Network” tool in the software was utilized to analyze the degree value of the nodes. The size and darkness of the point correspond to the degree of the node, and each node in the network represents a protein (Figure 2F). The top 10 candidate targets were prioritized from the PPI network using the Degree method in the Cytoscape plugin cytoHubba (Figure 2G). These analyses prioritized PIK3R1, HSP90AA1, SRC, AKT1, STAT3, PIK3CD, PIK3CB, EGFR, GRB2, and ESR1 as candidate core targets of chaetomugilin Q in breast cancer (Figure 2H). These targets are computationally prioritized and require experimental validation to confirm direct binding and functional relevance.

### 2.2. Results of GO and KEGG Enrichment Analysis

**Figure 3:**
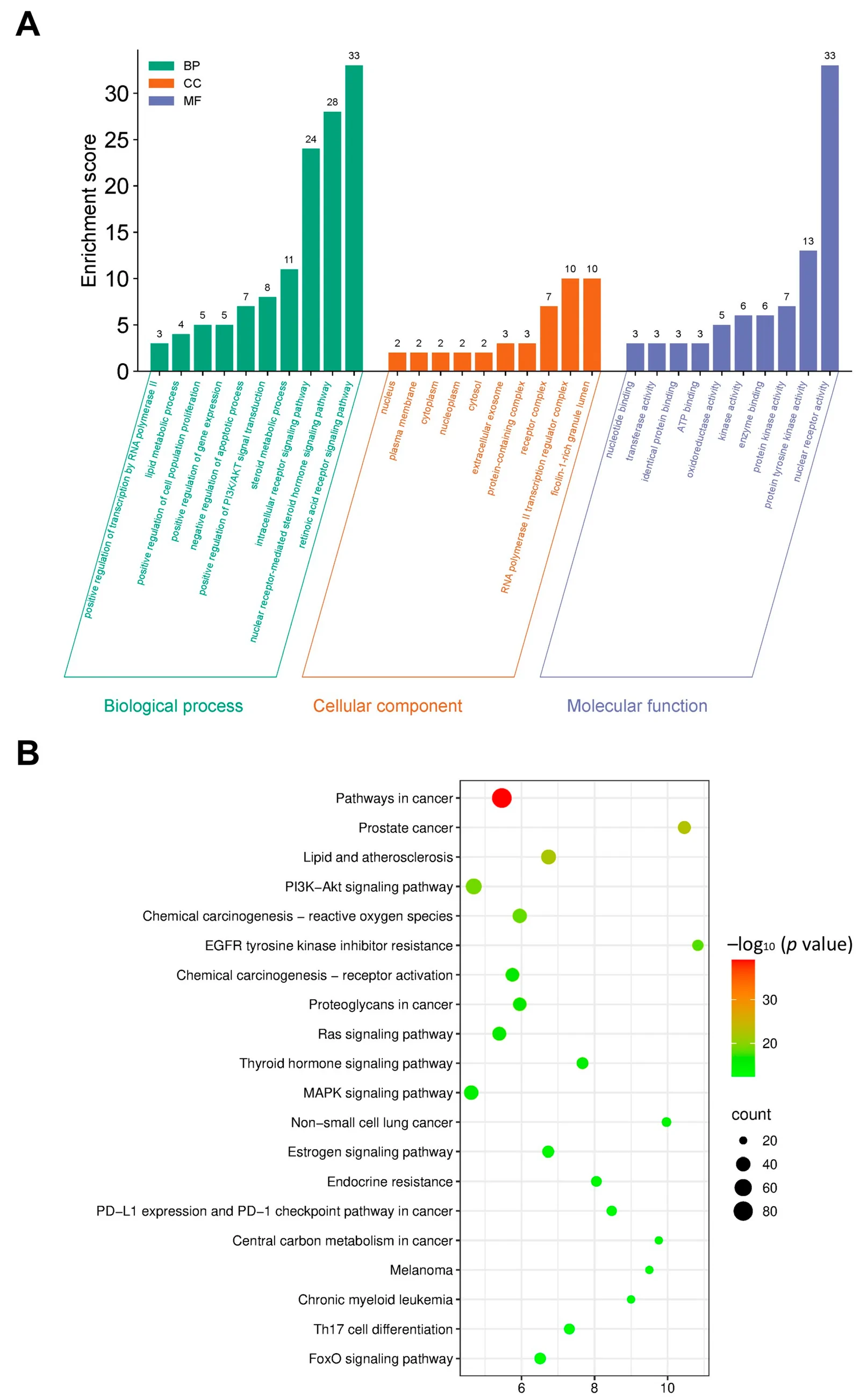
GO enrichment analysis of the 299 predicted targets of chaetomugilin Q in breast cancer (**A**). KEGG enrichment analysis of the 299 putative targets of chaetomugilin Q in breast cancer (**B**).

GO and KEGG enrichment analyses were performed on the 299 candidate targets. GO enrichment analysis yielded 1174 terms, including 839 terms associated with biological processes (BP), 100 terms associated with cellular components (CC), and 235 terms associated with molecular functions (MF). The top 10 terms in each category were selected for visualization using a bioinformatics tool (Figure 3A). The putative targets were enriched in BP terms, including negative regulation of apoptotic process, lipid metabolic process, positive regulation of gene expression, steroid metabolic process, positive regulation of phosphatidylinositol 3-kinase/protein kinase B signal transduction, positive regulation of transcription by RNA polymerase II, positive regulation of cell population proliferation, nuclear receptor-mediated steroid hormone signaling pathway, intracellular receptor signaling pathway, and retinoic acid receptor signaling pathway; CC terms, including cytosol, cytoplasm, extracellular exosome, nucleoplasm, nucleus, RNA polymerase II transcription regulator complex, ficolin-1-rich granule lumen, receptor complex, protein-containing complex, and plasma membrane; and MF terms, including nuclear receptor activity, protein kinase activity, kinase activity, protein tyrosine kinase activity, ATP binding, enzyme binding, transferase activity, identical protein binding, oxidoreductase activity, and nucleotide binding. These results indicate that the 299 predicted targets are associated with the identified BP, CC, and MF terms. However, they do not demonstrate that chaetomugilin Q directly regulates the corresponding biological processes or molecular functions, or affects the localization or assembly of the corresponding cellular components.

In the KEGG pathway enrichment analysis, 169 signaling pathways were identified, and the top 20 signaling pathways were selected for visualization (Figure 3B). The enriched pathways included Pathways in cancer, Prostate cancer, Lipid and atherosclerosis, PI3K-Akt signaling pathway, Chemical carcinogenesis–reactive oxygen species, EGFR tyrosine kinase inhibitor resistance, Chemical carcinogenesis–receptor activation, Proteoglycans in cancer, Ras signaling pathway, Thyroid hormone signaling pathway, MAPK signaling pathway, Non-small cell lung cancer, Estrogen signaling pathway, Endocrine resistance, PD-L1 expression and PD-1 checkpoint pathway in cancer, Central carbon metabolism in cancer, Melanoma, Chronic myeloid leukemia, Th17 cell differentiation, FoxO signaling pathway, among others. These results indicate that the 299 putative targets are enriched in multiple cancer-related signaling pathways. However, the KEGG enrichment analysis is based solely on the candidate targets and does not demonstrate that chaetomugilin Q directly regulates these pathways. Therefore, whether chaetomugilin Q functionally modulates these targets and pathways remains to be determined experimentally.

**Figure 4:**
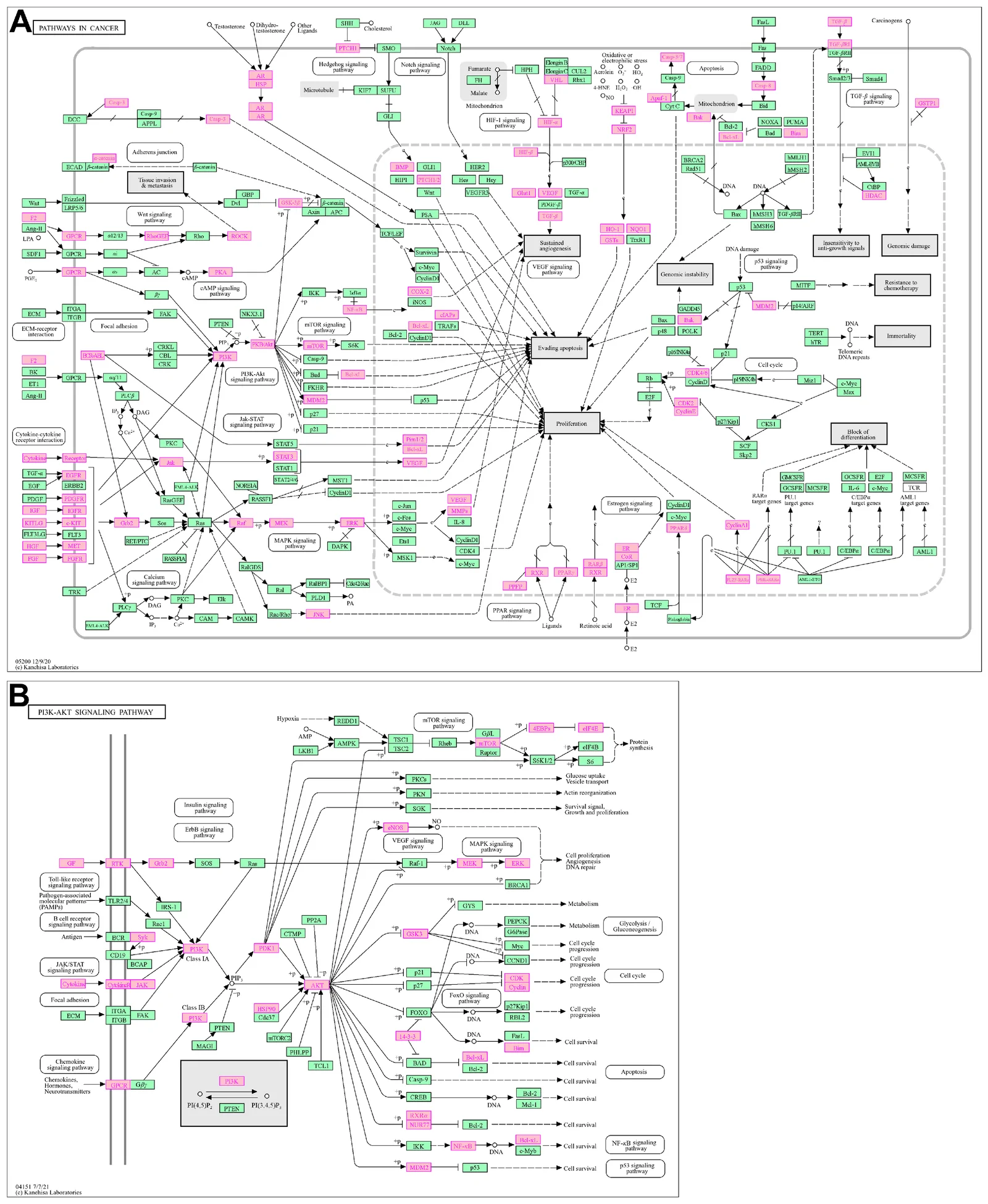
Pathways in cancer (hsa05200) (**A**). PI3K-Akt signaling pathway (hsa04151) (**B**). The KEGG pathway maps were generated using the KEGG database. Pathways are displayed with *Homo sapiens* (hsa) as the reference organism. Green boxes represent human-specific gene/enzyme nodes annotated in KEGG. Pink boxes indicate predicted targets mapped to the pathway. Boxes with both green and pink coloring represent predicted targets that are also annotated as human-specific genes/enzymes. White boxes indicate pathway nodes without such annotations. Arrows represent activation or molecular interactions; T-shaped lines represent inhibition; dashed lines represent indirect or unconfirmed interactions. Circles represent small molecules, ions, or metabolites.

According to the KEGG enrichment results, Pathways in cancer and the PI3K-Akt signaling pathway were selected for pathway mapping [34]. The pink markers in Figure 4A,B indicate the candidate targets mapped onto these pathways. These mappings visualize the potential pathway associations of the candidate targets; however, they do not demonstrate that chaetomugilin Q directly binds to or functionally modulates these targets or pathways. Therefore, these results should be considered hypothesis-generating and require further experimental validation.

### 2.3. Compound–Target–Pathway–Disease Network

**Figure 5:**
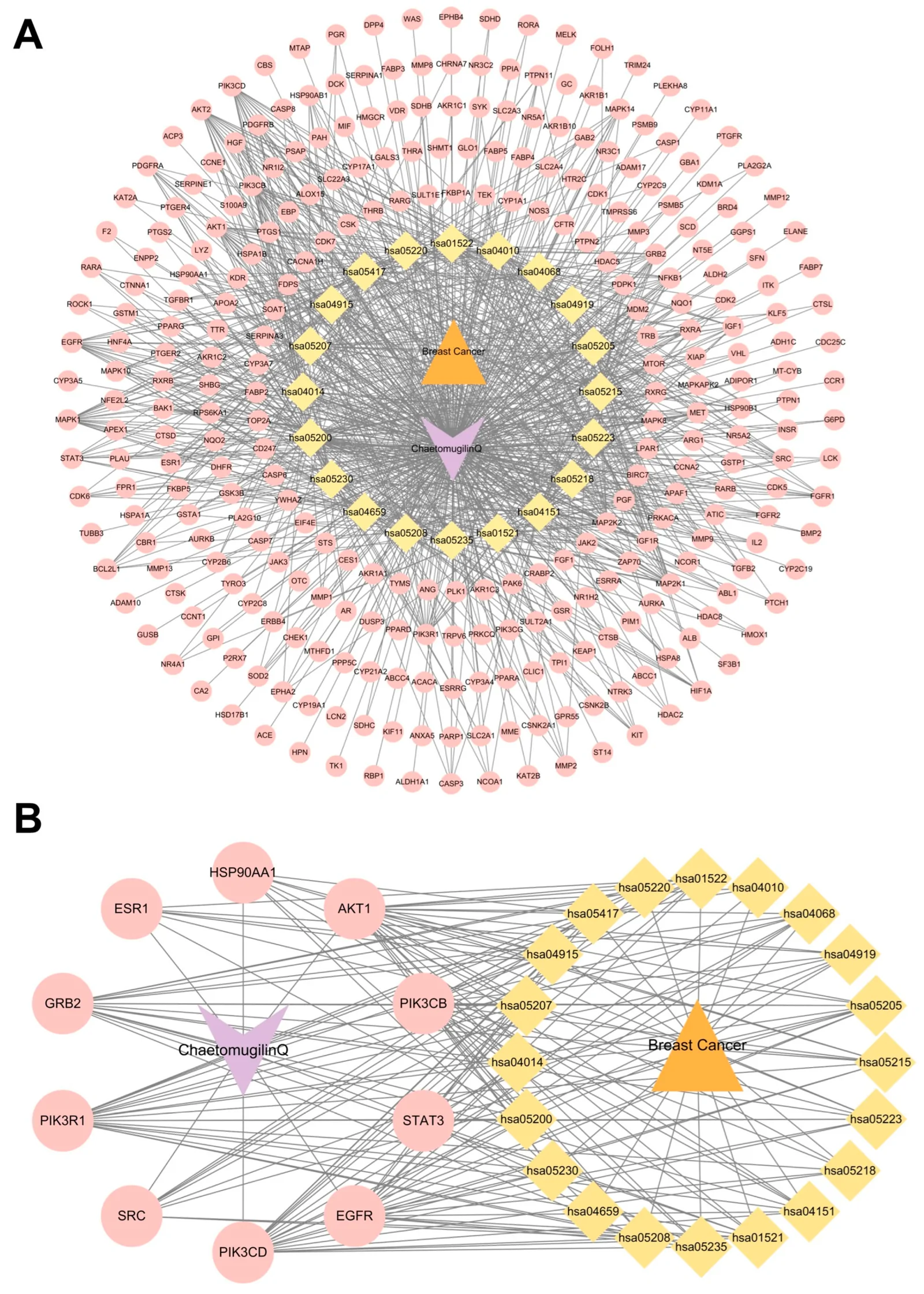
Network diagram of putative targets and pathways of Chaetomugilin Q in breast cancer (**A**). Core network diagram of predicted targets and pathways of Chaetomugilin Q in breast cancer (**B**). In the network, the orange triangle represents breast cancer; the purple V-shaped symbol represents chaetomugilin Q; the pink circles represent candidate targets; the yellow diamonds represent KEGG pathways (hsa IDs); and the gray lines represent interactions/associations among them.

A compound–target–pathway–disease network for chaetomugilin Q and breast cancer was constructed by importing the top 20 enriched KEGG pathways into Cytoscape 3.10.1. In Figure 5A,B, chaetomugilin Q, breast cancer, targets, and pathways are represented by a V shape, a triangle, circles, and diamonds, respectively. This network visualizes the predicted associations among chaetomugilin Q, candidate targets, enriched pathways, and breast cancer. The network suggests that chaetomugilin Q may be associated with multiple candidate targets and pathways.

### 2.4. Molecular Docking Prediction of Chaetomugilin Q with Candidate Core Targets

**Figure 6:**
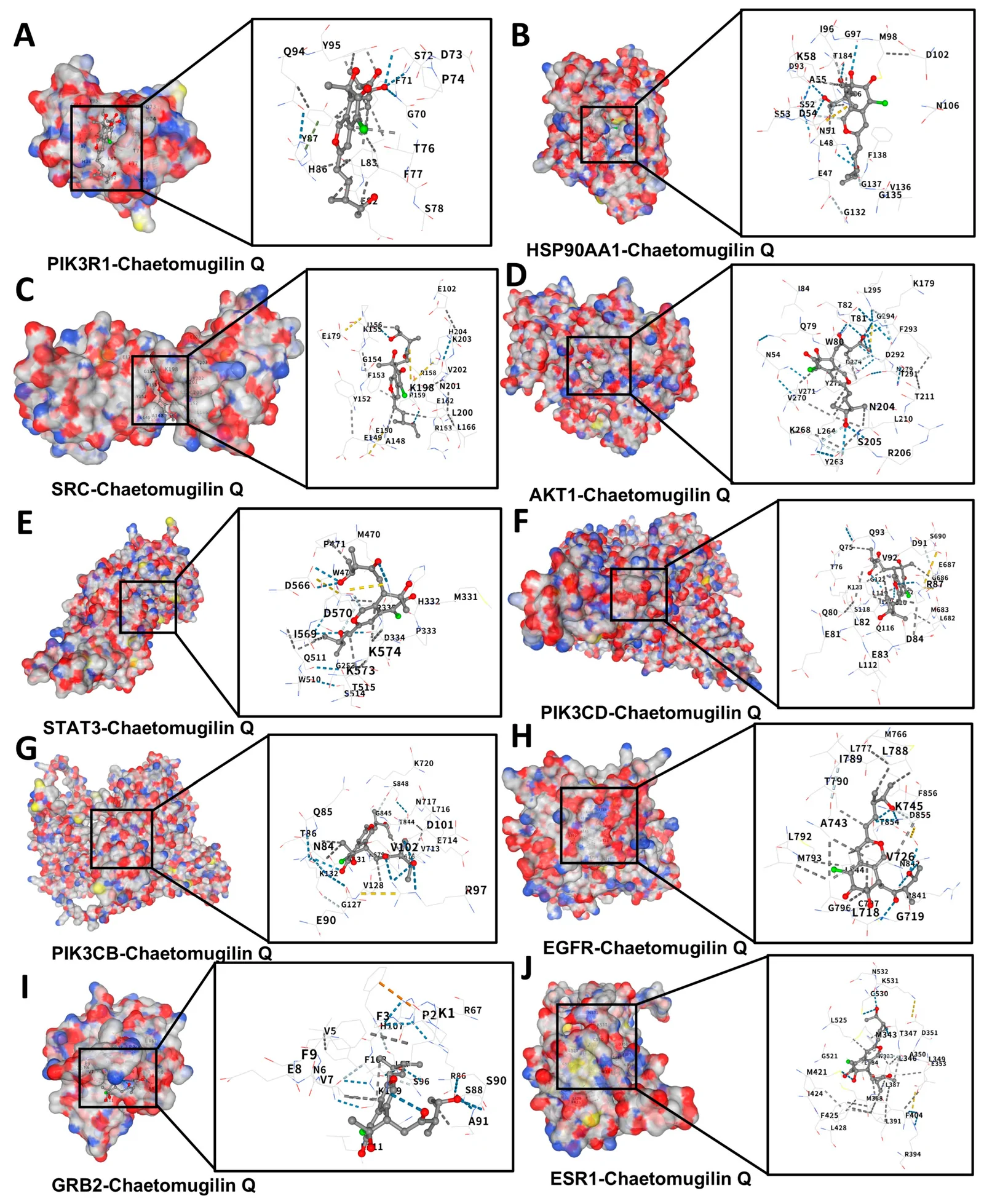
Predicted binding modes of chaetomugilin Q with candidate core targets. (**A**) PIK3R1 (PDB: 2IUG): overall binding pose (**left**) and predicted interactions with key residues such as Y95, G70 and T76 (**right**); (**B**) HSP90AA1 (PDB: 1OSF): overall binding surface (**left**) and predicted interactions with key residues such as G97, G137 and L48 (**right**); (**C**) SRC (PDB: 1A09): overall binding mode (**left**) and predicted interactions with key residues such as E150, K198 and G154 (**right**); (**D**) AKT1 (PDB: 7NH5): overall binding pose (**left**) and predicted interactions with key residues such as S205, T81 and N204 (**right**); (**E**) STAT3 (PDB: 6NJS): overall binding surface (**left**) and predicted interactions with key residues such as I569, D566 and K574 (**right**); (**F**) PIK3CD (PDB: 6PYR): overall binding mode (**left**) and predicted interactions with key residues such as R87, Q75 and D84 (**right**); (**G**) PIK3CB (AlphaFold DB: AF-P42338-F1): overall binding pose (**left**) and predicted interactions with key residues such as V128, D101 and N84 (**right**); (**H**) EGFR (PDB: 3W2S): overall binding surface (**left**) and predicted interactions with key residues such as G719, L788 and A743 (**right**); (**I**) GRB2 (PDB: 1BMB): overall binding mode (**left**) and predicted interactions with key residues such as A91, V7 and R86 (**right**); and (**J**) ESR1 (PDB: 1XP1): overall binding pose (**left**) and predicted interactions with key residues such as M388, L346 and C530 (**right**). The protein surface is colored by atom type (red, oxygen; blue, nitrogen; gray, carbon; yellow, sulfur). The gray stick model represents chaetomugilin Q. Amino acid residues are shown as thin lines, and dashed lines indicate non-covalent interactions (e.g., hydrogen bonds and hydrophobic contacts). The green sphere represents the chlorine atom in chaetomugilin Q, and the red spheres represent oxygen atoms. The black boxes highlight the predicted binding pockets (magnified views), with key residues annotated.

To predict the potential binding modes and interactions between chaetomugilin Q and the candidate core targets, molecular docking prediction was performed using CB-Dock2. CB-Dock2 is a docking tool for protein-ligand blind docking that integrates curvature-based cavity detection, AutoDock Vina-based molecular docking, and homologous template-based blind docking. Five possible binding cavities were identified, and the pose with the lowest predicted docking score was selected based on the Vina docking score. A Vina score of ≤−5.0 kcal/mol was used only as an empirical cutoff to prioritize docking poses for visual inspection. The predicted docking scores of chaetomugilin Q to the candidate core targets were less than −6.1 kcal/mol (Figure S1), and the predicted binding conformations of chaetomugilin Q with these candidate core targets are shown in Figure 6. These results suggest potential binding modes; however, they do not confirm direct binding or functional target engagement, both of which remain to be experimentally validated.

### 2.5. Clinical Association Analysis of Candidate Core Targets

#### 2.5.1. mRNA Expression Levels of Candidate Core Targets

**Figure 7:**
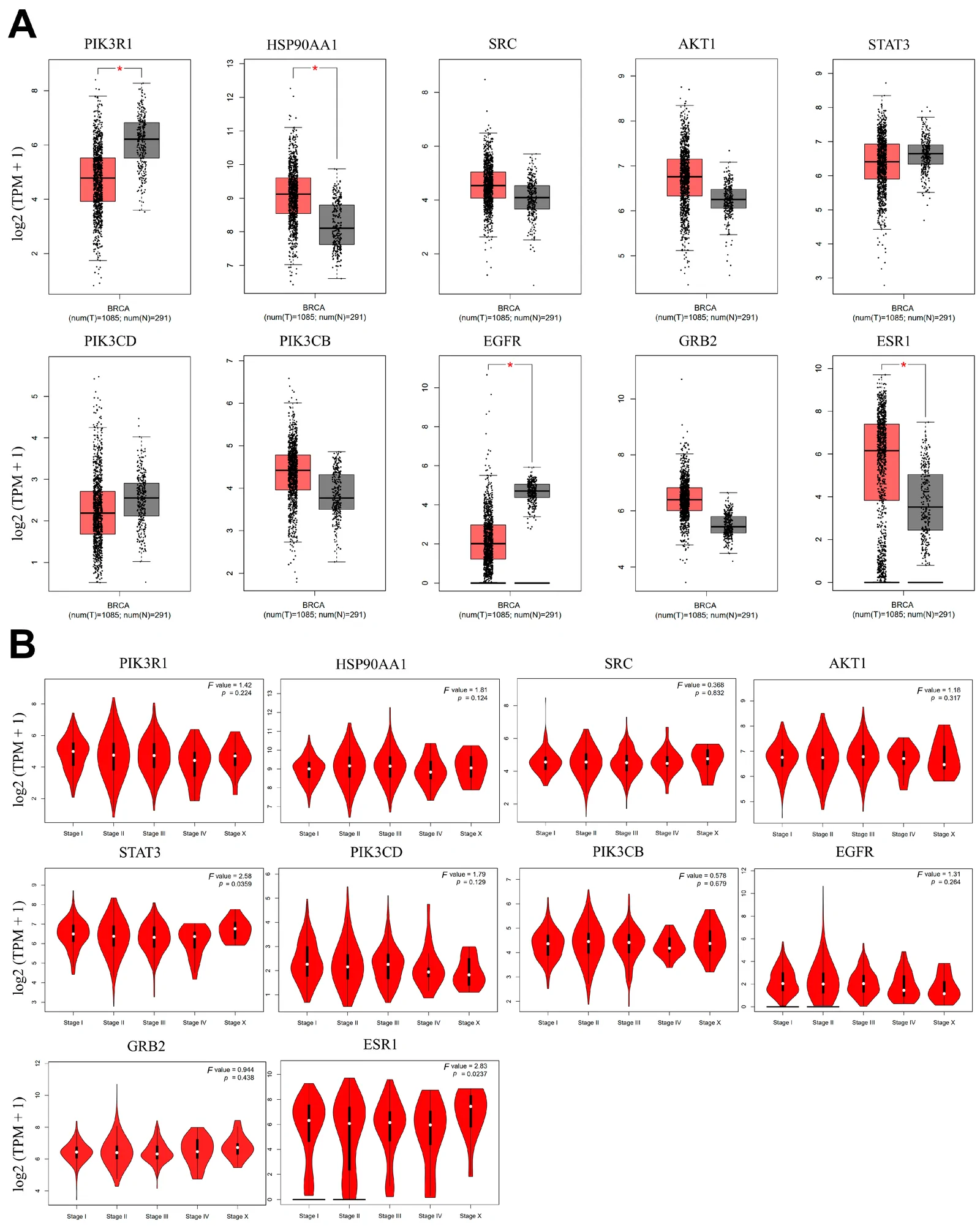
Box plot of the mRNA expression levels of candidate core targets (**A**). Red represents tumor tissues and gray represents normal tissues. In the box plots, the central line represents the median, the box represents the interquartile range (IQR), and the whiskers extend to the minimum and maximum values within 1.5 × IQR. Dots represent the expression values of individual samples. The red asterisk (*) indicates a statistically significant difference between the tumor and normal groups (*p* < 0.05). Relationship between the mRNA expression levels of candidate core targets and pathological stages in breast cancer patients (**B**). In the violin plots, the expression of candidate core targets is shown across different pathological stages of breast cancer, including Stage I–IV and unknown stage (Stage X). The black central line represents the median. The *F*-value and *p*-value from the ANOVA test are indicated.

The mRNA expression levels of the candidate core targets differed between breast cancer tissues and normal breast tissues (Figure 7A). The mRNA levels of ESR1 and HSP90AA1 were significantly higher in breast cancer tissues than in normal breast tissues, whereas those of EGFR and PIK3R1 were markedly lower in the former than in the latter (*p* < 0.01). The relationship between the mRNA expression of these candidate core targets and the pathological stage of breast cancer was also analyzed (Figure 7B). The mRNA levels of ESR1 and STAT3 were significantly associated with pathological stage (*p* < 0.05). These findings are based on analyses using GEPIA and indicate the potential associations.

#### 2.5.2. Protein Expression Levels of Candidate Core Targets

**Figure 8:**
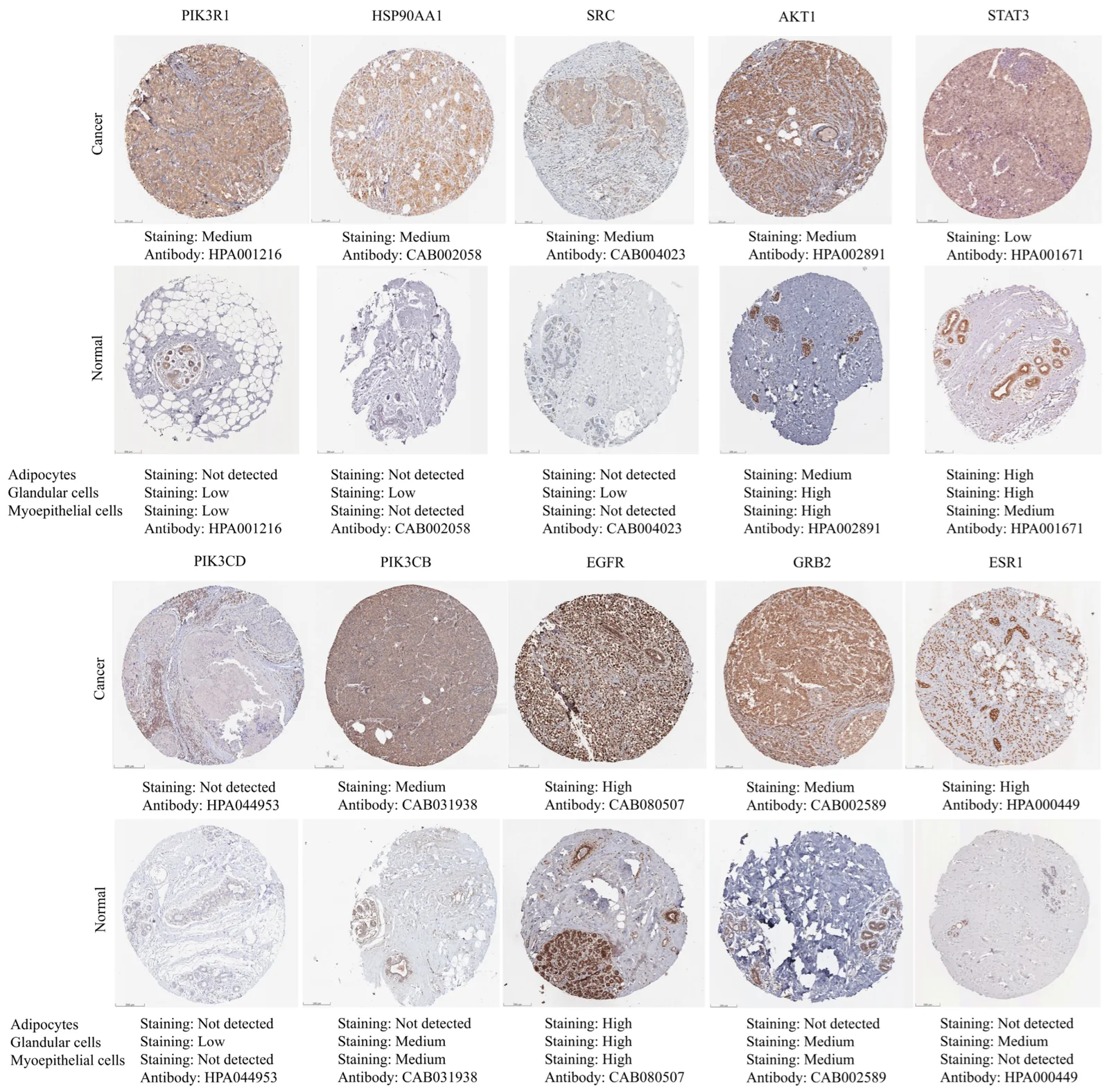
Immunohistochemical images of candidate core targets in normal and breast cancer patients from the HPA database. Scale bar, 200 μm.

The protein expression of the candidate core targets in breast cancer was examined by analyzing immunohistochemical staining images from the Human Protein Atlas (HPA) database (Figure 8). Compared with normal breast tissues, breast cancer tissues showed increased expression of ESR1, PIK3CB, GRB2, PIK3R1, SRC, and HSP90AA1 but decreased expression of STAT3. Moreover, EGFR and AKT1 showed high or moderate expression in both breast cancer tissues and normal breast tissues, whereas PIK3CD was rarely detected in either tissue type. These results are based on analyses using the HPA database and reveal the expression patterns of the candidate core targets.

#### 2.5.3. Genetic Alterations in Candidate Core Targets

**Figure 9:**
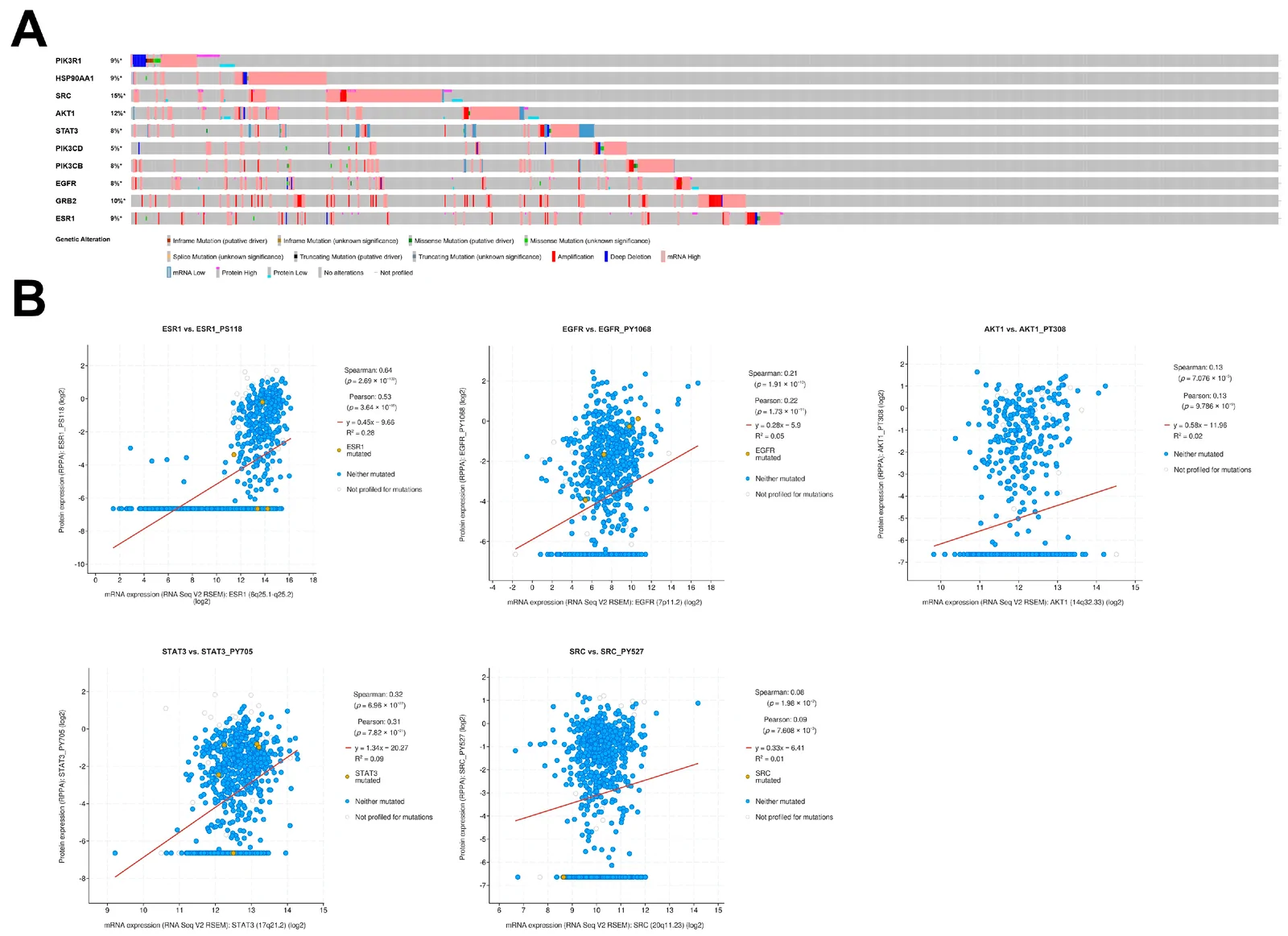
Information on the types of gene mutations in candidate core targets for breast cancer patients (**A**). Correlation between the mRNA and protein levels of ESR1, EGFR, AKT1, STAT3, and SRC (**B**). * The percentage was calculated based on samples with available data for each alteration type shown; not all samples were profiled for every alteration type.

624 of 1101 breast cancer patients (57%) had genetic mutations in the candidate core targets (Figure 9A). Furthermore, the protein levels of ESR1, EGFR, AKT1, STAT3, and SRC were positively correlated with their corresponding mRNA levels (Figure 9B). No correlation data were available for PIK3CB, GRB2, PIK3R1, PIK3CD, or HSP90AA1. These findings are based on analyses using the cBioPortal database and reveal genetic alterations in the candidate core targets and correlations between their mRNA and protein expression.

#### 2.5.4. Survival Analysis of Candidate Core Targets

**Figure 10:**
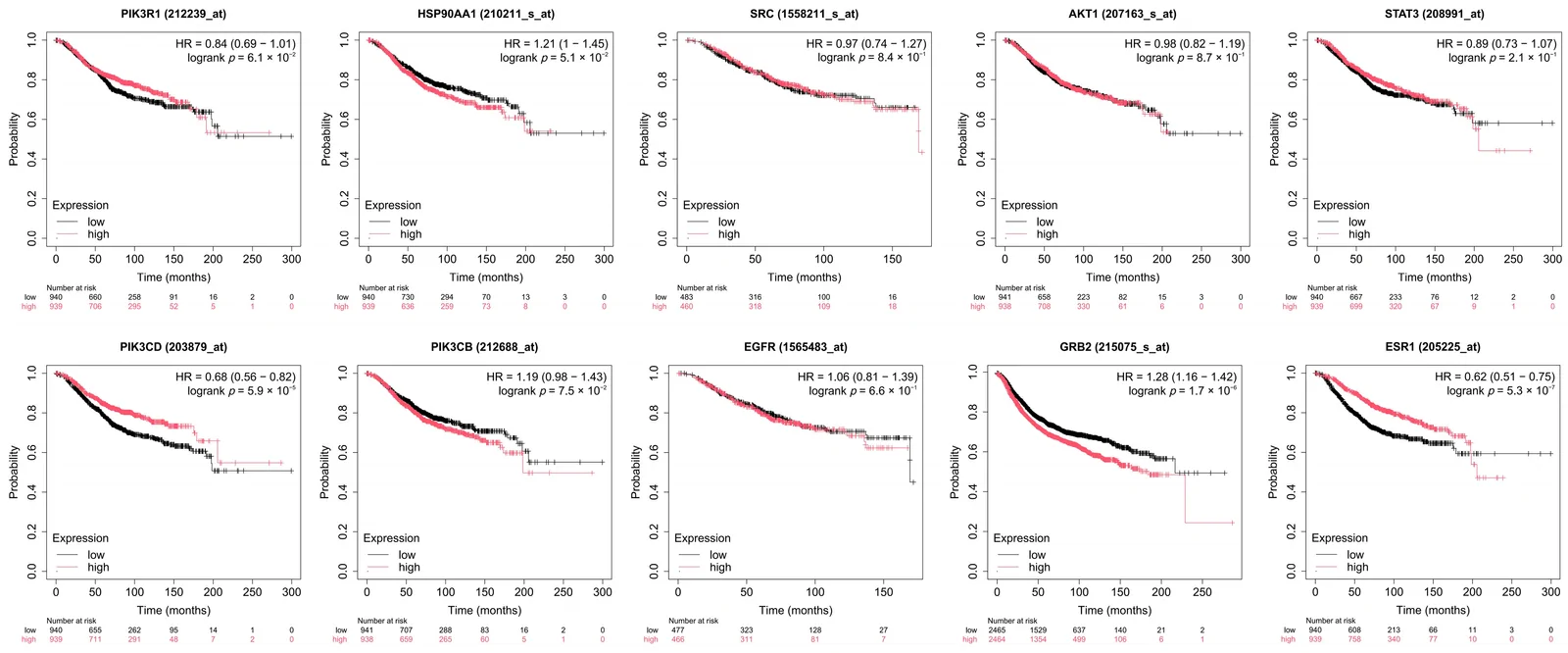
Relationship between the expression levels of candidate core targets and survival outcome in patients with breast cancer.

Kaplan–Meier analysis was used to examine the associations between the expression of the candidate core targets and the survival outcome of breast cancer patients. High expression of ESR1 and PIK3CD was associated with a better prognosis than low expression, whereas high expression of GRB2 was associated with a poorer prognosis than low expression (Figure 10). These survival associations reflect the prognostic relevance of the candidate core targets but should not be interpreted as evidence that chaetomugilin Q directly alters patient survival.

#### 2.5.5. Immune Cell Infiltration of Candidate Core Targets

**Figure 11:**
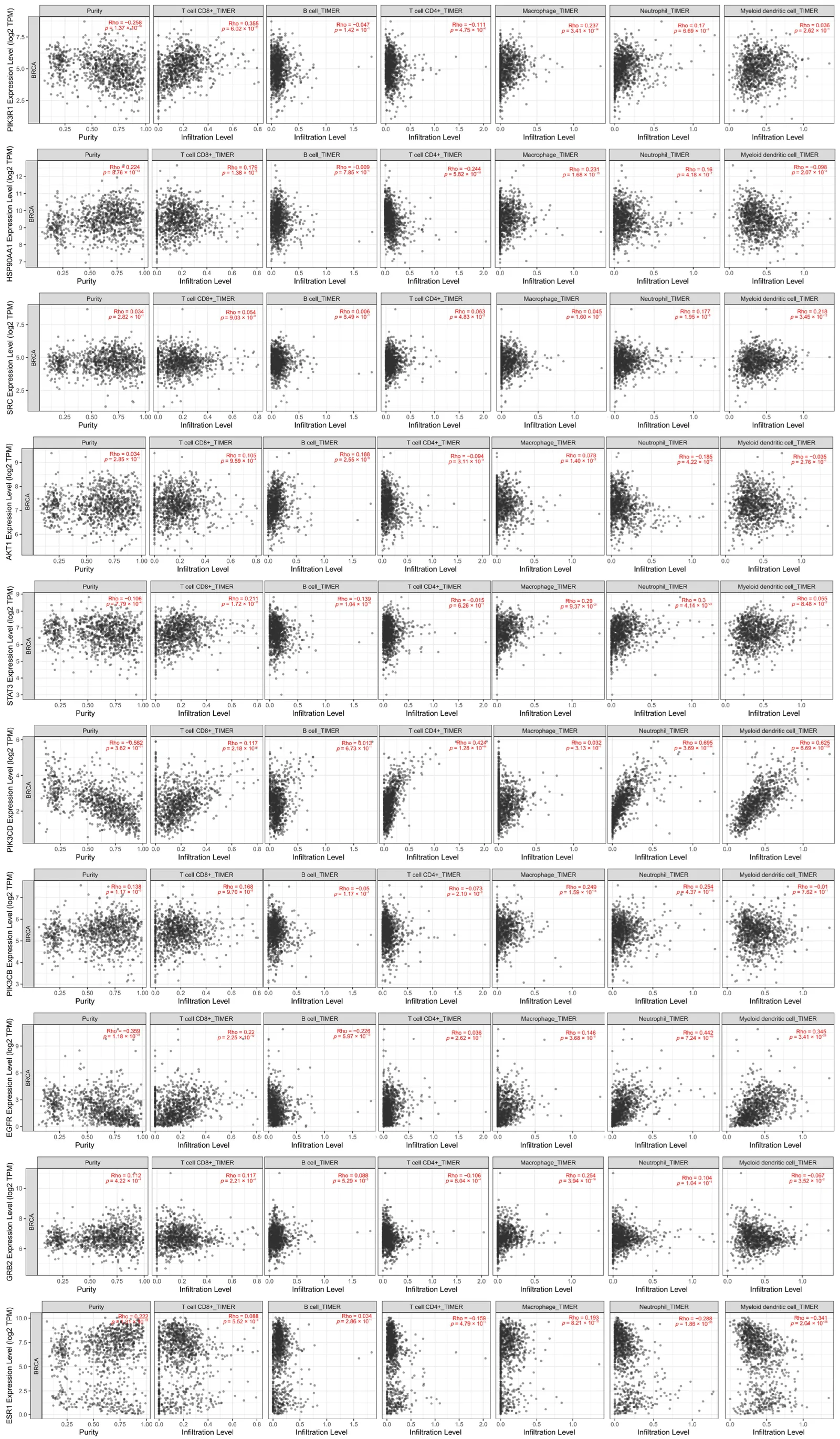
Associations between the expression of candidate core targets and immune cell infiltration in breast cancer.

The associations between the expression of the candidate core targets and immune cell infiltration in breast cancer were analyzed using the TIMER database (Figure 11). The results showed that PIK3R1 expression was positively correlated with the infiltration of CD8+T cells, macrophages, and neutrophils, and negatively correlated with the infiltration of CD4+T cells and with tumor purity. No correlation was observed between PIK3R1 expression and the infiltration of B cells or myeloid dendritic cells. HSP90AA1 expression was positively correlated with the infiltration of CD8+T cells, macrophages, and neutrophils, and with tumor purity, and negatively correlated with the infiltration of CD4+T cells and myeloid dendritic cells. No correlation was observed between HSP90AA1 expression and the infiltration of B cells. SRC expression was positively correlated with the infiltration of neutrophils, CD4+T cells, and myeloid dendritic cells. No correlation was observed between SRC expression and the infiltration of CD8+T cells, B cells, or macrophages, nor between SRC expression and tumor purity. AKT1 expression was positively correlated with the infiltration of CD8+T cells, B cells, and macrophages, and negatively correlated with the infiltration of CD4+T cells and neutrophils. No correlation was observed between AKT1 expression and the infiltration of myeloid dendritic cells, nor between AKT1 expression and tumor purity. STAT3 expression was positively correlated with the infiltration of CD8+T cells, macrophages, and neutrophils, and negatively correlated with the infiltration of B cells and with tumor purity. No correlation was observed between STAT3 expression and the infiltration of CD4+T cells or myeloid dendritic cells. PIK3CD expression was positively correlated with the infiltration of CD8+T cells, CD4+T cells, neutrophils, and myeloid dendritic cells, and negatively correlated with tumor purity. No correlation was observed between PIK3CD expression and the infiltration of B cells or macrophages. PIK3CB expression was positively correlated with the infiltration of CD8+T cells, macrophages, and neutrophils, and with tumor purity, and negatively correlated with the infiltration of CD4+T cells. No correlation was observed between PIK3CB expression and the infiltration of B cells or myeloid dendritic cells. EGFR expression was positively correlated with the infiltration of CD8+T cells, macrophages, neutrophils, and myeloid dendritic cells, and negatively correlated with the infiltration of B cells and with tumor purity. No correlation was observed between EGFR expression and the infiltration of CD4+T cells. GRB2 expression was positively correlated with the infiltration of CD8+T cells, B cells, macrophages, and neutrophils, and with tumor purity, and negatively correlated with the infiltration of CD4+T cells and myeloid dendritic cells. ESR1 expression was positively correlated with the infiltration of CD8+T cells and macrophages, and with tumor purity, and negatively correlated with the infiltration of CD4+T cells, neutrophils, and myeloid dendritic cells. No correlation was observed between ESR1 expression and the infiltration of B cells. These associations with immune cell infiltration reflect the immunological context of the candidate core targets. Whether chaetomugilin Q modulates immune cell infiltration remains to be experimentally investigated.

The analyses described above were performed using publicly available databases to provide clinical and biological context for the candidate core targets. However, these analyses do not demonstrate that chaetomugilin Q directly binds to and regulates these targets, or that these targets mediate its effects on gene expression, prognosis, or immune cell infiltration in breast cancer. Therefore, further experimental validation is required to confirm these proposed roles.

### 2.6. In Vitro Biological Activity and AR Pathway Assessment

To computationally examine the relationship between androgen receptor (AR) and the candidate core targets of chaetomugilin Q, we performed further bioinformatics analyses. The results suggested that AR may serve as a predicted hub node in the PPI network of the candidate core targets, particularly HSP90AA1, SRC, STAT3, and ESR1 (Figure S2A). Based on KEGG pathway enrichment analysis, Prostate cancer was selected for pathway mapping. As shown in Figure S2B, the AR signaling pathway may be closely associated with the PI3K/AKT and MAPK pathways. The AR signaling pathway is a central driver of prostate cancer and represents a crucial therapeutic target. Additionally, we computationally explored the potential mechanisms of chaetomugilin Q against prostate cancer using network pharmacology (Figures S3–S6). Molecular docking predicted a possible binding mode between chaetomugilin Q and AR, with a predicted docking score of −7.8 kcal/mol (Figure S7A), and the predicted binding mode is shown in Figure S7B.

To assess the antiproliferative effects of chaetomugilin Q on cancer cells, cell viability was evaluated using the MTS assay. The results showed that chaetomugilin Q significantly suppressed the proliferation of several cancer cell lines, including HepG2, Huh7, A375, B16F10, Reh, CCRF-CEM, NB4, Kasumi-1, PC-3, and MDA-MB-231. The cell viability curves suggested that chaetomugilin Q dose-dependently inhibited the proliferation of prostate and breast cancer cells (Figure S8A). The MTS assay showed that chaetomugilin Q exhibited IC_50_ values of 11.71 ± 1.22 µM and 8.83 ± 1.71 µM in PC-3 and MDA-MB-231 cells, respectively. Moreover, in AR-positive prostate cancer cells, chaetomugilin Q significantly inhibited AR transcriptional activity and markedly reduced prostate-specific antigen (PSA) mRNA expression in a dose-dependent manner. The AR inhibitory activity of chaetomugilin Q was greater than that of the selective AR inhibitors EPI-001 and MDV3100 in 22Rv1 and LNCaP cells (Figure S8B). These in vitro results demonstrate that chaetomugilin Q exerts antiproliferative activity in PC-3 and MDA-MB-231 cells, whereas it inhibits AR signaling in AR-positive 22Rv1 and LNCaP cells. Therefore, they do not establish that AR mediates the anticancer effects of chaetomugilin Q in breast cancer, nor do they validate the predicted core targets or pathways in breast cancer. Nevertheless, chaetomugilin Q warrants further investigation as a potential therapeutic candidate, and additional breast cancer-specific mechanistic studies are required.

### 2.7. Predicted ADMET Properties of Chaetomugilin Q

**Figure 12:**
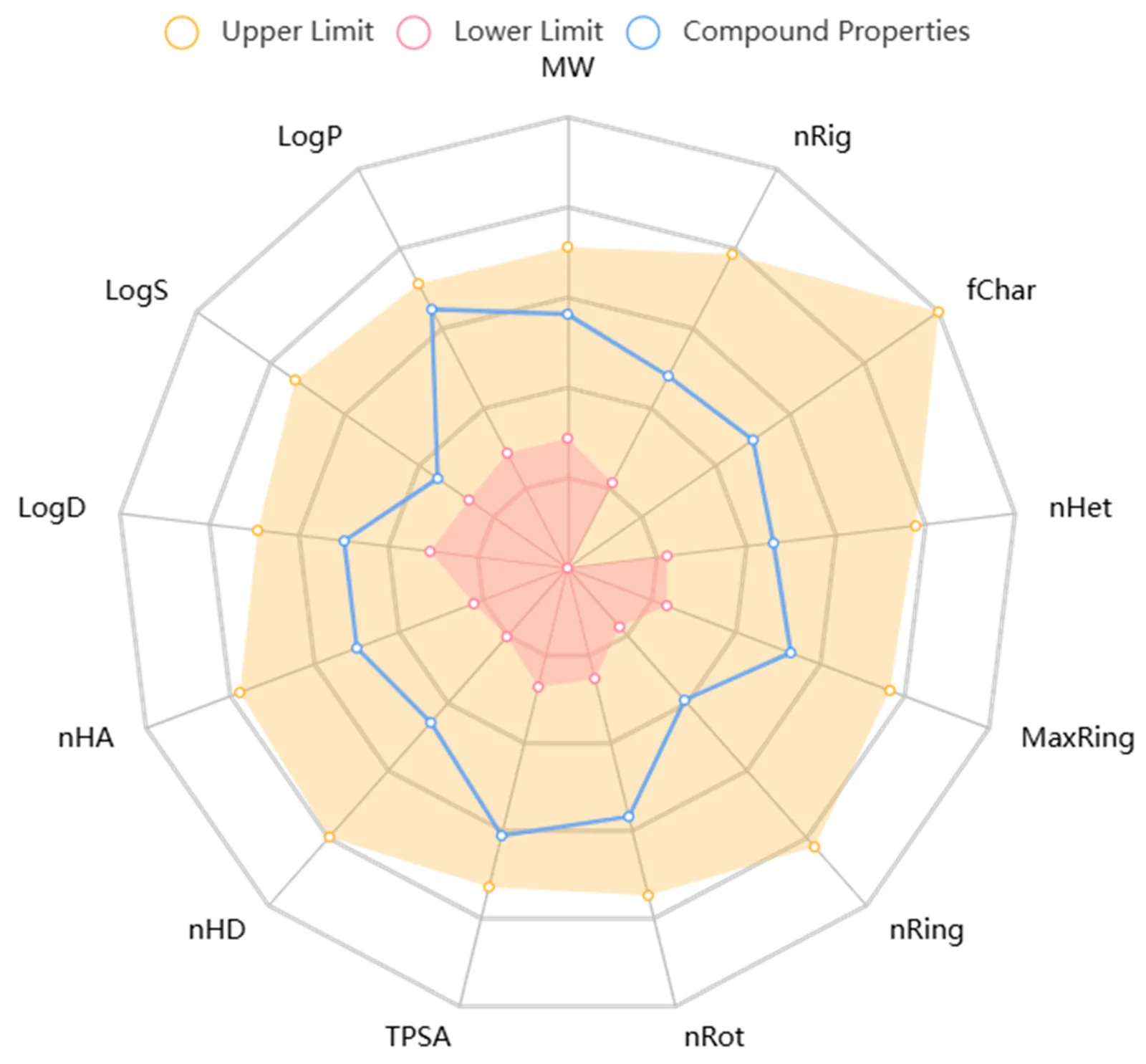
In silico ADMET profile of chaetomugilin Q.

To evaluate the drug-likeness of chaetomugilin Q, the ADMET profile (absorption, distribution, metabolism, excretion, and toxicity) was predicted using ADMETlab 2.0 [35]. The calculated molecular properties included molecular weight (MW), the logarithm of the n-octanol/water partition coefficient (LogP), the logarithm of aqueous solubility (LogS), the apparent distribution coefficient (LogD), the number of hydrogen-bond acceptors (nHA) and donors (nHD), the topological polar surface area (TPSA), the number of rotatable bonds (nRot), the number of rings (nRing), the maximum ring size (MaxRing), the number of heteroatoms (nHet), the formal charge (fChar), and the number of rigid bonds (nRig). As shown in Figure 12, the predicted minimum and maximum limits are depicted by the red and yellow radar charts, respectively. Compound properties that deviate from this range suggest suboptimal predicted physicochemical characteristics. All parameters of chaetomugilin Q predicted by the model fell within the drug-likeness range, suggesting favorable drug-like properties. However, these results are based on computational predictions and cannot replace experimental ADMET evaluation; further experimental validation is required to confirm the pharmacokinetic and toxicity profiles of chaetomugilin Q.

## 3. Discussion

Breast cancer is an exceptionally aggressive, highly heterogeneous, and therapeutically challenging cancer type. Currently, the therapeutic options for patients with breast cancer are limited to surgery, immunotherapy, chemotherapy, and radiotherapy [36]. Anthracyclines, taxanes, and platinum-based drugs remain the main systemic treatment options for patients with metastatic breast cancer [37]. Nevertheless, the absence of critical molecular targets contributes to its resistance to chemotherapy, causing limited survival for patients with the disease [38]. Therefore, there is an urgent need for the development of innovative and effective breast cancer treatment strategies. Chaetomugilin Q has shown weak to moderate cytotoxic activity against L1210, NCI-H187, P388, HL-60, and Vero cells [26,27]. In this study, chaetomugilin Q significantly suppressed the proliferation of several cancer cell lines, such as liver cancer, melanoma, leukemia, prostate cancer, and breast cancer cells. The aim of this study was to computationally explore the potential mechanisms underlying the antiproliferative effects of chaetomugilin Q in breast cancer using network pharmacology and molecular docking prediction, and to prioritize candidate core targets for future experimental validation.

KEGG enrichment analysis suggested that the putative targets of chaetomugilin Q in breast cancer were mainly enriched in Pathways in cancer, Prostate cancer, and the PI3K-Akt signaling pathway. The AR signaling pathway is an important target in AR-positive breast cancer subtypes [39]. However, in the present study, chaetomugilin Q inhibited AR signaling in AR-positive prostate cancer cells. Since these experiments were not performed in AR-positive breast cancer cells, they do not demonstrate that AR mediates the antiproliferative effects of chaetomugilin Q in breast cancer. The phosphoinositide 3-kinase (PI3K)/protein kinase B (Akt) signaling pathway is commonly hyperactivated in cancers and contributes to cell survival, growth, proliferation, metastasis, and angiogenesis of breast cancer, making it a significant therapeutic target [40]. Currently, a large number of studies are evaluating the PI3K-Akt signaling pathway inhibitors, such as alpelisib, capivasertib, and ipatasertib [9]. Nevertheless, numerous resistance mechanisms to PI3K-Akt signaling pathway inhibitors have emerged, leading to their limited efficacy against breast cancer [41]. Combination strategies consisting of PI3K-Akt inhibitors and other anti-neoplastic therapies provide a promising approach to overcome treatment resistance [42]. Collectively, these pathway enrichment results suggest that multiple signaling pathways may be involved. However, whether chaetomugilin Q functionally modulates these pathways in breast cancer remains to be determined.

A network pharmacology-based analysis prioritized the 10 candidate core targets: PIK3R1, HSP90AA1, SRC, AKT1, STAT3, PIK3CD, PIK3CB, EGFR, GRB2, and ESR1. The p85 alpha, p55 alpha, and p50 alpha (PIK3R1) are regulatory subunits of PI3K. The p110 beta (PIK3CB) and p110delta (PIK3CD) are catalytic subunits of PI3K [43]. PI3K is a critical protein that mediates cell survival, proliferation, and metabolism, making it a promising therapeutic target for breast cancer [9]. As a cytoplasmic isoform of heat shock protein 90 (HSP90), HSP90 alpha class A member 1 (HSP90AA1) is a vital molecular chaperone that regulates the stability and activity of numerous oncogenic proteins [44]. Accordingly, targeting HSP90 alpha is a potential therapeutic strategy for patients with breast cancer. Proto-oncogene tyrosine-protein kinase Src (SRC), a non-receptor tyrosine protein kinase, plays a crucial role in cell growth, division, proliferation, differentiation, survival, adhesion, motility, drug resistance and stemness in tumor cells by regulating multiple signaling pathways [45]. Given its role in these functions, SRC kinase represents a promising molecular target for treating breast cancer [46]. Protein kinase B alpha (AKT1) is a serine-threonine protein kinase that regulates various cellular processes, including cell growth, proliferation, and apoptosis [47]. AKT1 has also emerged as an important target for the treatment of breast cancer [9]. Signal transducer and activator of transcription 3 (STAT3), a transcriptional factor, plays an essential role in modulating important processes such as proliferation, differentiation, migration, invasion, angiogenesis, growth, survival, apoptosis, cancer cell stemness, tumor-promoting inflammation, anti-tumor immune response, metabolic reprogramming, and acquired drug resistance [48]. Owing to its pivotal functions, STAT3 has become a significant therapeutic target in breast cancer [49]. The surface of cancer cells overexpresses epidermal growth factor receptor (EGFR), a tyrosine kinase receptor, which is responsible for proliferation, growth, adhesion, invasion, migration, and survival [50]. However, due to EGFR mutations, anti-EGFR therapies have shown limited efficacy against breast cancer in preclinical and clinical studies [51]. Despite the presence of mutations, EGFR remains a promising and actionable therapeutic target in breast cancer. Growth factor receptor-bound protein 2 (GRB2) is an adaptor protein that plays a pivotal role in growth, proliferation, metastasis, and autophagy of breast cancer cells, making it an attractive therapeutic target [52,53]. Estrogen receptor alpha (ESR1), a nuclear hormone receptor, is often low or absent in TNBC but highly expressed in nearly 70% of breast cancers [54]. ESR1 mutation, an emerging clinical biomarker, represents a main mechanism of acquired resistance to hormonal therapy [55,56]. Therefore, ESR1 may contribute to the broad breast cancer-related network identified in this study, but it is unlikely to be the primary mediator in TNBC. Taken together, these observations suggest that these proteins may be candidate targets of chaetomugilin Q in breast cancer. However, neither direct binding of chaetomugilin Q to these proteins nor functional dependence on them has been experimentally demonstrated. Hence, these proteins should be regarded as predicted or putative targets rather than experimentally confirmed targets.

This study has some limitations. First, the poor accuracy and completeness of the databases may have impacted the credibility of the selected genes. The present study focuses on breast cancer as a whole, with particular emphasis on TNBC as one of the most aggressive and therapeutically challenging subtypes. The network pharmacology analysis was performed using breast cancer-related targets without subtype restriction, and antiproliferative activity was evaluated in MDA-MB-231 TNBC cells. However, the other cancer cell lines tested in this study were used only to evaluate the broad antiproliferative activity of chaetomugilin Q and were not used to support subtype-specific conclusions. Therefore, the conclusions of this study are intended to apply to breast cancer broadly, with potential relevance to TNBC, and the underlying findings should be further validated in subtype-specific contexts. Although the public database analyses provide useful clinical and biological context for the candidate core targets, they are correlative in nature and do not demonstrate direct binding or regulation by chaetomugilin Q. The mRNA and protein expression, mutation, survival, and immune-infiltration associations should therefore be interpreted as target-level associations rather than compound-induced effects. Direct target engagement and functional regulation remain to be experimentally validated, ideally using approaches such as biochemical binding assays, target knockdown or overexpression, pathway perturbation, and in vivo models. Second, MDA-MB-231 is an AR-low TNBC cell line, and PC-3 is also an AR-low prostate cancer cell line. Therefore, the antiproliferative effects observed in these cells are unlikely to be mediated by AR signaling. The AR luciferase reporter assays and PSA qPCR analysis were performed in AR-positive prostate cancer cell lines, namely, 22Rv1 and LNCaP. These results indicate that chaetomugilin Q can modulate AR transcriptional activity in these prostate cancer cell lines, but they do not confirm AR as a functional mediator of the effects of chaetomugilin Q in breast cancer, nor do they validate the predicted core targets or pathways in breast cancer. Although the antiproliferative activity of chaetomugilin Q was supported by MTS dose–response assays and IC_50_ determination in the tested cancer cell lines, direct experimental evidence for the binding of chaetomugilin Q to the candidate core targets and for its functional modulation of the associated signaling pathways is still lacking. Therefore, these predicted targets and pathways require further experimental validation. Third, because this study used multi-target blind docking without a validated active site or a predefined grid box for each target, the CB-Dock2 results should be regarded as first-pass computational predictions for hypothesis generation rather than as active-site-specific or confirmatory evidence of binding. Accordingly, the predicted compound-target interactions are described as potential binding modes rather than as confirmed active-site binding. The molecular docking results provide only preliminary computational predictions of potential binding modes but do not confirm direct binding of chaetomugilin Q to the candidate core targets or functional target engagement by chaetomugilin Q. Moreover, neither redocking of co-crystallized ligands with RMSD calculation nor experimental binding assays were performed, and no positive-control ligands or known inhibitors were included as benchmarks for the docking analyses. Therefore, these docking results should be regarded as hypothesis-generating rather than confirmatory, and require further experimental validation. Although the predicted docking scores suggest potentially favorable binding modes, they do not confirm stable binding. Because no MD simulations were performed, these scores alone are not sufficient to establish stable binding or active-site occupancy. Despite these constraints, this study provides a theoretical basis and a hypothesis-generating framework for further exploration of chaetomugilin Q in breast cancer.

## 4. Materials and Methods

### 4.1. Databases and Software

The databases and software employed in this investigation are presented in Table 1.

### 4.2. Computational Prediction of Chaetomugilin Q Targets and Breast Cancer-Related Targets

Depending on the input requirements of each tool, either the canonical SMILES string or the 3D SDF structure of chaetomugilin Q was used as input for target prediction in SuperPred, PharmMapper, and GalaxySagittarius-AF [57,58,59]. A total of 97, 299, and 828 entries were obtained from SuperPred, PharmMapper, and GalaxySagittarius-AF, respectively. For the GalaxySagittarius-AF results, entries with a score ≥ 0.269 were retained (*n* = 197), whereas all entries from SuperPred and PharmMapper were retained without filtering. Subsequently, the UniProt IDs corresponding to all entries obtained from SuperPred, PharmMapper, and GalaxySagittarius-AF were converted into gene names using the UniProt database [60], and duplicate gene names were removed, yielding 96, 292, and 208 predicted targets, respectively. The target genes obtained from the three databases were combined and deduplicated in Microsoft Excel to obtain the predicted targets of chaetomugilin Q. The target prediction data for chaetomugilin Q were obtained in May 2025.

The GeneCards, OMIM, CTD, DrugBank, and TTD databases were searched for breast cancer-related targets using “breast cancer” as the search term [61,62,63,64,65]. A total of 50,000 entries were obtained from GeneCards, and genes with a relevance score of ≥10 were retained (*n* = 4559). A total of 444 entries were obtained from OMIM; after removal of duplicate gene names, 169 genes remained. The numbers of genes obtained from CTD, DrugBank, and TTD were 122, 201, and 101, respectively. The target genes collected from the five databases were merged and deduplicated in Microsoft Excel to obtain breast cancer-related targets. The data on breast cancer-related targets were collected in September 2025.

### 4.3. Screening for Overlapping Candidate Targets Between Chaetomugilin Q Targets and Breast Cancer-Related Targets

Venny 2.1.0 was used to intersect the predicted targets of chaetomugilin Q with breast cancer-related targets, and the overlapping candidate targets were obtained and visualized using a Venn diagram [66]. These overlapping targets were regarded as candidate targets of chaetomugilin Q in breast cancer for subsequent network pharmacology analysis. However, this intersection represents a computational prediction and does not provide evidence that chaetomugilin Q directly targets these genes or that they mediate its therapeutic effects.

### 4.4. PPI Network Construction and Candidate Core Target Prioritization

The overlapping candidate targets of chaetomugilin Q and breast cancer were imported into the STRING database to construct a PPI network [67]. The species was set to “Homo sapiens”, the minimum required interaction score was set to the highest confidence (0.900), and disconnected nodes were hidden. For further analysis, the TSV files were imported into Cytoscape 3.10.1 for visualization [68]. Using the cytoHubba plugin in Cytoscape 3.10.1, the top 10 candidate core targets were prioritized using the Degree function [69]. These targets were computationally prioritized and require experimental validation to confirm direct binding of chaetomugilin Q to them and to establish their functional relevance.

### 4.5. GO and KEGG Enrichment Analysis

GO and KEGG enrichment analyses of the candidate targets of chaetomugilin Q in breast cancer were performed using the Functional Annotation module in DAVID [70]. The enrichment results were organized and sorted in Microsoft Excel, and the top 10 or 20 terms from each analysis were selected for visualization using a bioinformatics platform [71]. These analyses reveal the functional enrichment of the candidate targets and do not demonstrate direct regulation of these targets by chaetomugilin Q.

### 4.6. Construction of Compound–Target–Pathway–Disease Network

The compound–target–pathway–disease network was constructed in Cytoscape 3.10.1 by integrating chaetomugilin Q, its candidate targets for breast cancer, the top 20 enriched KEGG pathways, and breast cancer, all represented as nodes. The edges represent the predicted associations among these nodes. This network visualizes predicted relationships and does not demonstrate direct molecular interactions or experimentally validated mechanisms.

### 4.7. Molecular Docking Prediction Between Chaetomugilin Q and Candidate Core Targets

Molecular docking was performed using CB-Dock2 to predict potential intermolecular interactions and binding modes between chaetomugilin Q and the candidate core targets prioritized in this study [72]. The 3D structure of chaetomugilin Q was retrieved from the PubChem database in SDF format, while the PDB files of the candidate core target proteins were downloaded from the RCSB PDB database for PIK3R1 (2IUG), HSP90AA1 (1OSF), SRC (1A09), AKT1 (7NH5), STAT3 (6NJS), PIK3CD (6PYR), EGFR (3W2S), GRB2 (1BMB), and ESR1 (1XP1) [73,74]. For PIK3CB (UniProt accession P42338), the AlphaFold DB model AF-P42338-F1 was used because no experimental PDB structure with adequate coverage of the relevant domains was available. This model was obtained from the AlphaFold Protein Structure Database rather than the RCSB PDB. The receptor structures were submitted to the CB-Dock2 server (accessed November 2025), which performed automated receptor preparation and blind docking using the AutoDock Vina engine (version 1.2.0). CB-Dock2 combines cavity detection, docking, and homologous template fitting to improve the accuracy of binding site identification and pose prediction. By default, water molecules, co-crystallized ligands, and other hetero groups (including ions and cofactors) were removed. Hydrogen atoms were added to the receptor, and the prepared receptor was converted to PDBQT format. The ligand was automatically prepared by adding hydrogens and converting it into a suitable 3D format for docking. Missing residues and missing heavy atoms were not modeled; only the heavy atoms present in the submitted structural models were used, apart from the added hydrogens. All parameters were set to default unless otherwise specified. The predicted binding pose with the lowest Vina docking score was selected for further analysis. The predicted intermolecular interactions were visualized in 3D. These docking results are computational predictions and do not confirm direct binding or functional target engagement.

### 4.8. In Vitro Antiproliferative Activity and AR Pathway Analysis

#### 4.8.1. Cell Lines and Culture

Human breast cancer cells (MDA-MB-231), human prostate cancer cells (PC-3, 22Rv1, and LNCaP), human liver cancer cells (HepG2 and Huh-7), human acute leukemia cells (Reh, CCRF-CEM, NB4, and Kasumi-1), human malignant melanoma cells (A375), and mouse melanoma cells (B16-F10) were purchased from the Cell Bank of the Chinese Academy of Sciences or the American Type Culture Collection (ATCC). PC-3 cells were cultured in Ham’s F-12K medium (Gibco; Thermo Fisher Scientific, Grand Island, NY, USA) supplemented with 10% fetal bovine serum (FBS; Gibco; Thermo Fisher Scientific, Grand Island, NY, USA) and 1% penicillin/streptomycin (P/S; Gibco; Thermo Fisher Scientific, Grand Island, NY, USA). 22Rv1, LNCaP, Reh, CCRF-CEM, and NB4 cells were cultured in RPMI 1640 medium (Gibco; Thermo Fisher Scientific, Grand Island, NY, USA) supplemented with 10% FBS and 1% P/S. Kasumi-1 cells were maintained in RPMI 1640 medium (Gibco; Thermo Fisher Scientific, Grand Island, NY, USA) supplemented with 20% FBS and 1% P/S. MDA-MB-231, HepG2, Huh-7, A375, and B16-F10 cells were maintained in Dulbecco’s modified Eagle’s medium (DMEM; HyClone; HyClone Laboratories, Logan, UT, USA) supplemented with 10% FBS and 1% P/S. All cell lines were incubated at 37 °C in a humidified incubator with 5% CO_2_.

#### 4.8.2. Cell Viability Assay

Cell viability was measured by MTS assay (Promega; Promega Corporation, Madison, WI, USA) following the manufacturer’s instructions. Cells were seeded into 96-well plates at densities ranging from 2000 to 5000 cells per well. After overnight incubation, the cells were treated with chaetomugilin Q for 72 h. Subsequently, 20 μL MTS solution was added directly into each well, and the plates were incubated at 37 °C for 1–2 h. Absorbance was measured at 490 nm using a microplate reader.

#### 4.8.3. Dual-Luciferase Reporter Assay

22Rv1 cells were seeded in 24-well plates and co-transfected with the AR luciferase reporter plasmid pAR-TA-Luc (Beyotime; Beyotime Biotechnology, Shanghai, China) and a Renilla luciferase plasmid using Lipofectamine 2000 (Invitrogen; Life Technologies Corporation, Carlsbad, CA, USA). After 6 h of incubation, the transfected cells were stimulated with 10 nM R1881 (Sigma-Aldrich; Sigma-Aldrich Corporation, St. Louis, MO, USA) and treated with chaetomugilin Q (2, 5, and 10 μM) for 16 h. The AR antagonists EPI-001 and MDV3100 were used as positive controls. Firefly and Renilla luciferase activities were measured using the Dual-Luciferase Reporter Assay System (Beyotime; Beyotime Biotechnology, Shanghai, China). Firefly luciferase activity was normalized to Renilla luciferase activity.

#### 4.8.4. Quantitative Real-Time Polymerase Chain Reaction

Serum-starved LNCaP cells were stimulated with R1881 and treated with chaetomugilin Q (1, 5, and 10 μM) for 12 h. Total RNA was extracted from LNCaP cells using TRIzol reagent (Takara; Takara Bio Inc., Kusatsu, Shiga, Japan). RNA was reverse-transcribed into complementary DNA (cDNA) using a cDNA Reverse Transcription Kit (Takara). Quantitative real-time polymerase chain reaction (qPCR) was performed using SYBR Green Master Mix (Applied Biosystems; Thermo Fisher Scientific, Waltham, MA, USA). mRNA expression levels were normalized to GAPDH expression. The primer sequences are listed in Table S1.

### 4.9. Independent Bioinformatic Assessment of Candidate Core Targets

#### 4.9.1. mRNA Expression Analysis of Candidate Core Targets

In the Expression DIY module of the GEPIA database, the mRNA expression levels and pathological staging of the candidate core targets were analyzed [75]. The |log2FC| cutoff was set at 1, and the *p*-value cutoff was set at 0.01. Subsequently, the mRNA expression levels of the candidate core targets in breast cancer tissues and normal breast tissues were examined, and their associations with pathological stages were evaluated. These analyses are based on data obtained using GEPIA and do not demonstrate that chaetomugilin Q directly regulates these targets.

#### 4.9.2. Protein Expression Analysis of Candidate Core Targets

To examine the protein expression patterns of the candidate core targets in breast cancer tissues, immunohistochemical data from the HPA database were analyzed [76]. Furthermore, the expression levels of these candidate core target proteins in breast cancer tissues were compared with those in normal breast tissues to identify potential differences in protein expression. This analysis is based on data from the HPA database and does not constitute experimental validation of direct regulatory effects of chaetomugilin Q on these targets.

#### 4.9.3. Genetic Alteration Analysis of Candidate Core Targets

The breast cancer dataset (TCGA, Firehose Legacy; 1896 samples) in the cBioPortal database was analyzed [77]. Information on genetic alterations and mutation sites in the candidate core targets was retrieved from cBioPortal. Associations between the mRNA and protein expression levels of the candidate core targets were also examined using the cBioPortal database. These analyses are based on data from the cBioPortal database and do not provide evidence for direct regulation of these targets by chaetomugilin Q.

#### 4.9.4. Prognostic Analysis of Candidate Core Targets

To examine the potential prognostic relevance of the candidate core targets in breast cancer, the Kaplan–Meier Plotter was used to assess the associations between the expression of the candidate core targets and patient survival [78]. Survival outcomes were compared between patients with high and low expression of each of the candidate core targets, and hazard ratios (HRs) and log-rank *p*-values were calculated. This analysis is based on data from the Kaplan–Meier Plotter and does not demonstrate that chaetomugilin Q directly modulates the expression or activity of these targets.

#### 4.9.5. Immune Cell Infiltration Analysis of Candidate Core Targets

To examine the potential associations between the expression of the candidate core targets and immune cell infiltration in breast cancer, we analyzed these potential associations using the TIMER database. The abundances of six immune cell populations (B cells, CD8+ T cells, CD4+ T cells, macrophages, neutrophils, and myeloid dendritic cells) were also estimated using TIMER [79]. This analysis is based on data obtained using TIMER and does not provide evidence that chaetomugilin Q directly regulates these targets or that it modulates immune cell infiltration.

### 4.10. Statistical Analysis

The differential expression analysis was performed using GEPIA with one-way ANOVA, with disease state or pathological stage as the grouping factor. The immunohistochemistry (IHC) results from the HPA database were analyzed using the Mann–Whitney U test. The log-rank test was employed using the Kaplan–Meier Plotter to compare survival curves, and HRs and log-rank *p*-values were calculated. The associations between the expression of the candidate core targets and immune cell infiltration were analyzed using Spearman’s correlation in TIMER. The correlations between the mRNA and protein expression levels of the candidate core targets were examined by calculating both Spearman’s and Pearson’s correlation coefficients using cBioPortal. Statistical analyses for in vitro experiments were performed using GraphPad Prism 8 software. Statistical significance was defined as a *p*-value of less than 0.05. These database analyses only describe associations and do not establish causal relationships or direct regulation of these targets by chaetomugilin Q.

## 5. Conclusions

In summary, we predicted and explored the potential mechanisms underlying the effects of chaetomugilin Q on breast cancer by integrating network pharmacology and molecular docking prediction. The results suggest that chaetomugilin Q may interact with the candidate core targets, including PIK3R1, HSP90AA1, SRC, AKT1, STAT3, PIK3CD, PIK3CB, EGFR, GRB2, and ESR1, and may be associated with pathways such as pathways in cancer, prostate cancer, and the PI3K-Akt signaling pathway. However, these findings are based on computational predictions and therefore require further experimental validation to confirm direct binding and functional target engagement. This study provides a theoretical basis for future studies on the underlying mechanisms of chaetomugilin Q against breast cancer.

## Figures and Tables

**Table 1:** Basic information of the databases and software involved in the study.

Name	URL
PubChem	https://pubchem.ncbi.nlm.nih.gov/ (accessed on 28 April 2025)
PharmMapper	https://www.lilab-ecust.cn/pharmmapper/ (accessed on 19 May 2025)
SuperPred	https://prediction.charite.de/index.php (accessed on 21 May 2025)
GalaxySagittarius-AF	https://galaxy.seoklab.org/sagittarius_af (accessed on 21 May 2025)
GeneCards	https://www.genecards.org/ (accessed on 18 September 2025)
CTD	https://ctdbase.org/ (accessed on 18 September 2025)
DrugBank	https://go.drugbank.com/ (accessed on 23 September 2025)
OMIM	https://www.omim.org/ (accessed on 18 September 2025)
TTD	https://ttd.idrblab.cn/ (accessed on 18 September 2025)
GEPIA	http://gepia.cancer-pku.cn/ (accessed on 7 November 2025)
The Human Protein Atlas	https://www.proteinatlas.org/ (accessed on 4 November 2025)
cBioPortal	https://www.cbioportal.org/ (accessed on 7 November 2025)
Kaplan–Meier Plotter	https://kmplot.com/analysis/ (accessed on 7 November 2025)
TIMER3	https://compbio.cn/timer3/ (accessed on 25 March 2026)
UniProt	https://www.uniprot.org/ (accessed on 24 May 2025)
STRING	https://cn.string-db.org/ (accessed on 17 March 2026)
RCSB PDB	https://www.rcsb.org/ (accessed on 7 November 2025)
DAVID	https://davidbioinformatics.nih.gov/ (accessed on 17 March 2026)
Bioinformatics	https://www.bioinformatics.com.cn/ (accessed on 17 March 2026)
Venny2.1.0	https://bioinfogp.cnb.csic.es/tools/venny/ (accessed on 16 March 2026)
KEGG Mapper	https://www.kegg.jp/kegg/mapper/ (accessed on 17 March 2026)
CB-Dock2	https://cadd.labshare.cn/cb-dock2/php/index.php (accessed on 7 November 2025)
Cytoscape 3.10.1	https://cytoscape.org/download.html (accessed on 16 March 2026)
GraphPad Prism 8	https://www.graphpad.com/features (accessed on 16 March 2026)
Microsoft Excel 2021	https://www.microsoft.com/zh-cn/microsoft-365/excel (accessed on 16 March 2026)

## Data Availability

The datasets utilized and/or analyzed in the investigation are available from the corresponding authors upon reasonable request.

## Supplementary Materials

The following supporting information can be downloaded at: https://www.mdpi.com/article/10.3390/molecules31203661/s1; Table S1: Primer sequences used for quantitative real-time polymerase chain reaction (qPCR). Figure S1: Predicted Vina docking scores of chaetomugilin Q with the candidate core targets. Figure S2: PPI network of AR and the candidate core targets (A). Prostate cancer (B). Figure S3: Collection of prostate cancer-related targets (A). Venn diagram of overlapping targets between chaetomugilin Q targets and prostate cancer-related targets (B). PPI network diagram of the candidate targets in prostate cancer (C). Visualization of the PPI network in prostate cancer (D). Top 10 candidate core targets in prostate cancer (E). Candidate core targets in prostate cancer ranked by degree centrality (F). Figure S4: GO enrichment analysis of the candidate targets of chaetomugilin Q in prostate cancer (A). KEGG enrichment analysis of the candidate targets of chaetomugilin Q in prostate cancer (B). Figure S5: Pathways in cancer (A). PI3K-Akt signaling pathway (B). Figure S6: Compound-candidate target–pathway–disease network (A). Compound-candidate core target–pathway–disease network (B). Figure S7: Predicted molecular docking results of chaetomugilin Q with the AR protein (PDB: 1T65) (A). Binding mode of chaetomugilin Q with the AR protein (B). Figure S8: Chaetomugilin Q significantly inhibits the proliferation of various cancer cell lines, including liver cancer (HepG2 and Huh7), melanoma (A375 and B16F10), leukemia (Reh, CCRF-CEM, NB4, and Kasumi-1), prostate cancer (PC-3), and breast cancer (MDA-MB-231) (A). Chaetomugilin Q markedly suppresses AR signaling in AR-positive prostate cancer cells (B). The two AR inhibitors, EPI-001 and MDV3100, were used as positive controls. The data are presented as means ± SD or means ± SEM (*n* = 3). Statistical significance is indicated as follows: * *p* < 0.05, ** *p* < 0.01, *** *p* < 0.001. ns, not significant.

## Abbreviations

TNBC: Triple Negative Breast Cancer; GO: Gene Ontology; KEGG: Kyoto Encyclopedia of Genes and Genomes; CTD: Comparative Toxicogenomics Database; OMIM: Online Mendelian Inheritance in Man; TTD: Therapeutic Target Database; DAVID: Database for Annotation, Visualization, and Integrated Discovery; STRING: Search Tool for the Retrieval of Interacting Genes/Proteins; PPI: Protein–Protein Interaction; GEPIA: Gene Expression Profiling Interactive Analysis; HPA: Human Protein Atlas; TIMER: Tumor Immune Estimation Resource; BP: Biological Progression; CC: Cellular Component; MF: Molecular Function; PR: Progesterone Receptor; ER: Estrogen Receptor; HER2: Human Epidermal Growth Factor Receptor-2; ESR1: Estrogen Receptor 1; EGFR: Epidermal Growth Factor Receptor; PIK3CB: Phosphatidylinositol-4,5-Bisphosphate 3-Kinase Catalytic Subunit Beta; AKT1: AKT Serine/Threonine Kinase 1; HSP90AB1: Heat Shock Protein 90 Alpha Family Class B Member 1; PIK3R1: Phosphoinositide-3-Kinase Regulatory Subunit 1; STAT3: Signal Transducer and Activator of Transcription 3; PIK3CD: Phosphatidylinositol-4,5-Bisphosphate 3-Kinase Catalytic Subunit Delta; SRC: SRC Proto-Oncogene, Non-Receptor Tyrosine Kinase; HSP90AA1: Heat Shock Protein 90 Alpha Family Class A Member 1; AR: Androgen Receptor; PSA: Prostate-Specific Antigen; SMILES: Simplified Molecular Input Line Entry System; MTS Assay: MTS Cell Proliferation Assay Kit; PDB: Protein Data Bank; TCGA: The Cancer Genome Atlas; qPCR: Quantitative Real-Time Polymerase Chain Reaction.
