# Supplementary material for: Exploring the Underlying Molecular Mechanisms of Chaetomugilin Q in Breast Cancer Using Network Pharmacology, Molecular Docking, and Bioinformatics
Source: Molecules. 2026 Oct 13;31(20):3661. doi: 10.3390/molecules31203661 (PMC13707441; doi:10.3390/molecules31203661)
Supplement: Supplementary file 1 [file molecules-31-03661-s001.zip › Supplementary material.pdf]

Supplementary Data

Table S1. Primer sequences used for quantitative real-time polymerase chain reaction (qPCR).

Gene	Primer sequences (5′-3′)	
PSA	Forward	CTTGTAGCCTCTCGTGGCAG
	Reverse	GACCTTCATAGCATCCGTGAG
GAPDH	Forward	CGACAGTCAGCCGCATCTT
	Reverse	CCGTTGACTCCGACCTTCA

CurPocket ID	Vina score	Cavity volume (Å^3)	Center (x, y, z)	Docking size (x, y, z)
C4	-6.8	97	8, 41, 12	23, 23, 23
C1	-6.1	190	7, 23, 18	23, 23, 23
C3	-5.8	151	12, 31, 35	23, 23, 23
C2	-5.6	153	16, 25, 15	23, 23, 23
C5	-5.5	75	0, 34, 18	23, 23, 23

PIK3R1-Chaetomugilin Q

CurPocket ID	Vina score	Cavity volume (Å^3)	Center (x, y, z)	Docking size (x, y, z)
C1	-7.8	729	74, -27, 63	23, 23, 23
C3	-6.2	135	64, -18, 58	23, 23, 23
C4	-5.9	129	65, -35, 52	23, 23, 23
C2	-5.8	148	69, -33, 45	23, 23, 23
C5	-5.8	77	51, -25, 68	23, 23, 23

HSP90AA1-Chaetomugilin Q

CurPocket ID	Vina score	Cavity volume (Å^3)	Center (x, y, z)	Docking size (x, y, z)
C1	-7.4	840	41, 13, 28	23, 23, 23
C4	-5.9	69	55, 21, 14	23, 23, 23
C2	-5.6	156	42, 10, 13	23, 23, 23
C3	-5.6	92	38, -5, 47	23, 23, 23
C5	-5.6	66	41, 12, 47	23, 23, 23

SRC-Chaetomugilin Q

CurPocket ID	Vina score	Cavity volume (Å^3)	Center (x, y, z)	Docking size (x, y, z)
C1	-8.7	5867	10, -14, -12	23, 35, 32
C2	-7.5	248	13, -27, -9	23, 23, 23
C3	-7.1	247	25, -27, -25	23, 23, 23
C5	-7.0	216	2, 3, -4	23, 23, 23
C4	-6.8	246	13, -2, 4	23, 23, 23

AKT1-Chaetomugilin Q

CurPocket ID	Vina score	Cavity volume (Å^3)	Center (x, y, z)	Docking size (x, y, z)
C1	-6.8	892	5, 32, 20	23, 23, 23
C2	-6.8	525	2, 26, 29	23, 23, 23
C4	-6.6	429	16, 12, 19	23, 23, 23
C3	-6.3	431	-1, 9, 26	23, 23, 23
C5	-4.6	347	-29, -27, 62	23, 23, 23

STAT3-Chaetomugilin Q

CurPocket ID	Vina score	Cavity volume (Å^3)	Center (x, y, z)	Docking size (x, y, z)
C1	-7.9	5998	34, 21, 1	23, 33, 35
C3	-7.8	3922	15, 25, 22	30, 23, 35
C2	-7.4	4502	25, 0, 7	29, 23, 31
C5	-7.3	2065	9, 6, 9	29, 23, 23
C4	-6.7	2123	-6, 1, 25	33, 23, 23

PIK3CD-Chaetomugilin Q

CurPocket ID	Vina score	Cavity volume (Å^3)	Center (x, y, z)	Docking size (x, y, z)
C1	-8.2	8642	-18, -3, -14	31, 35, 35
C4	-8.1	1545	21, 1, 6	23, 23, 23
C2	-7.4	7283	-7, -15, 17	32, 35, 35
C5	-7.1	1148	-13, 6, 9	23, 23, 23
C3	-6.9	2028	5, 6, -13	23, 23, 31

PIK3CB-Chaetomugilin Q

CurPocket ID	Vina score	Cavity volume (Å^3)	Center (x, y, z)	Docking size (x, y, z)
C1	-7.9	2545	1, 3, 7	23, 23, 32
C4	-6.9	168	1, 12, 0	23, 23, 23
C2	-6.8	287	17, 8, 13	23, 23, 23
C5	-6.6	131	-9, 5, 21	23, 23, 23
C3	-6.5	184	20, 0, 19	23, 23, 23

EGFR-Chaetomugilin Q

CurPocket ID	Vina score	Cavity volume (Å^3)	Center (x, y, z)	Docking size (x, y, z)
C1	-6.1	504	31, 30, 16	23, 23, 23
C5	-5.8	36	32, 9, 13	23, 23, 23
C3	-5.7	68	42, 18, 9	23, 23, 23
C4	-5.5	55	30, 23, 20	23, 23, 23
C2	-5.4	123	18, 12, 13	23, 23, 23

GRB2-Chaetomugilin Q

CurPocket ID	Vina score	Cavity volume (Å^3)	Center (x, y, z)	Docking size (x, y, z)
C1	-7.0	2235	28, -3, 22	35, 23, 23
C4	-6.6	193	14, 5, 36	23, 23, 23
C5	-6.5	166	32, 8, 17	23, 23, 23
C3	-5.7	204	29, 10, 4	23, 23, 23
C2	-5.3	330	27, 17, 33	23, 23, 23

ESR1-Chaetomugilin Q

Figure S1. Predicted Vina docking scores of chaetomugilin Q with the candidate core targets.

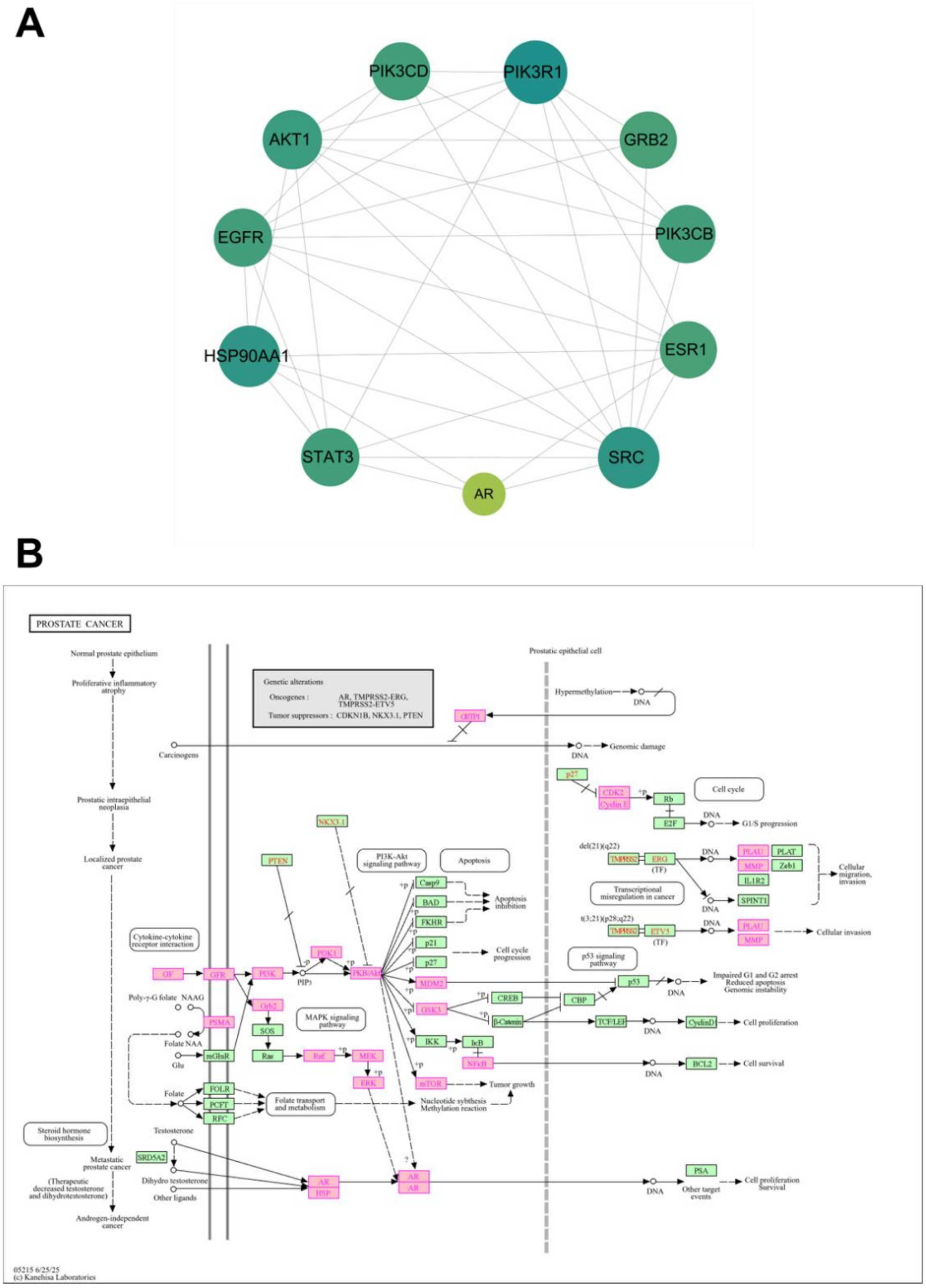

Figure S2. PPI network of AR and the candidate core targets (A). Prostate cancer (B).

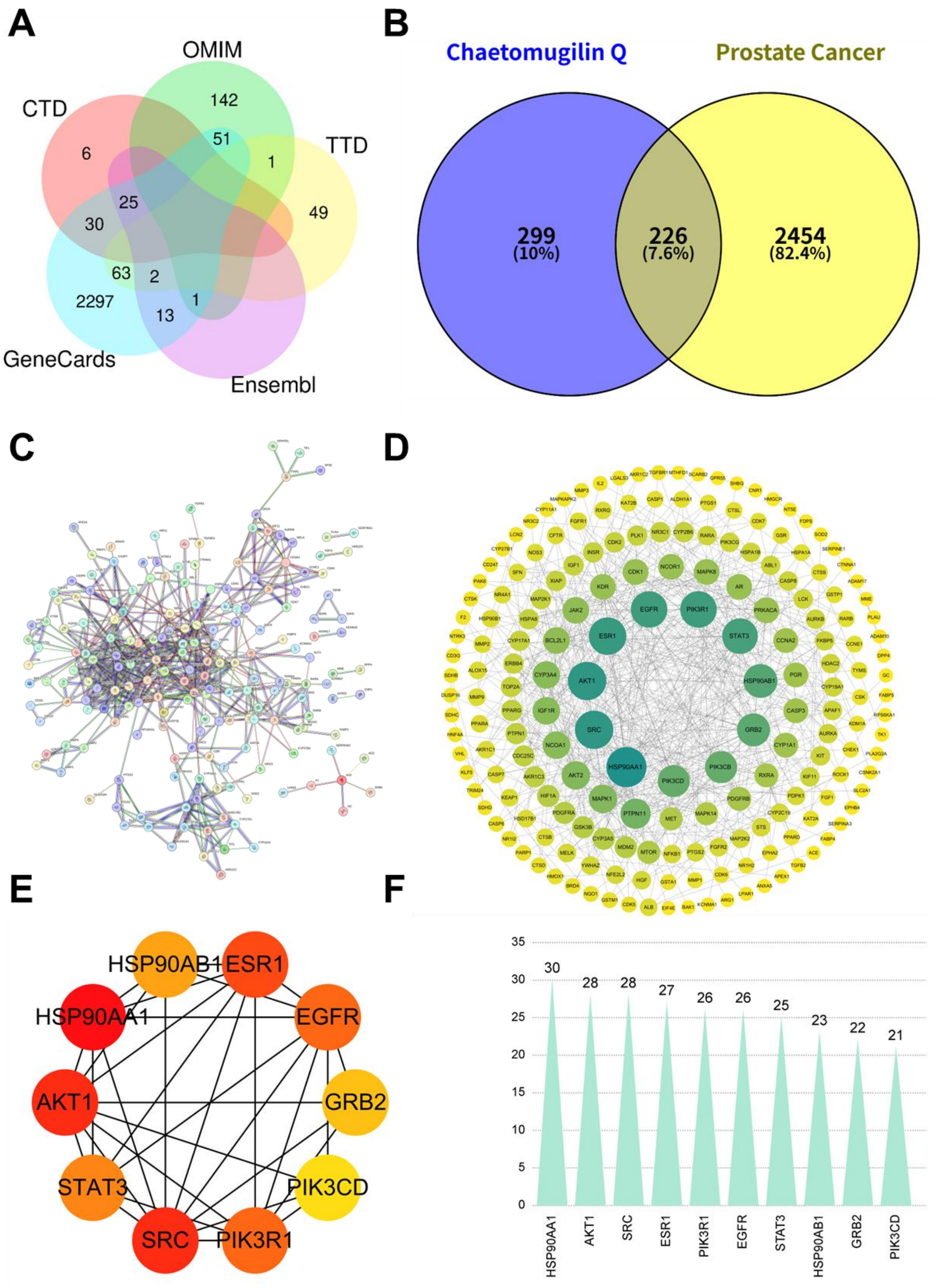

Figure S3. Collection of prostate cancer-related targets (A). Venn diagram of overlapping targets between chaetomugilin Q targets and prostate cancer-related targets (B). PPI network diagram of the candidate targets in prostate cancer (C). Visualization of the PPI network in prostate cancer (D). Top 10 candidate core targets in prostate cancer (E). Candidate core targets in prostate cancer ranked by degree centrality (F).

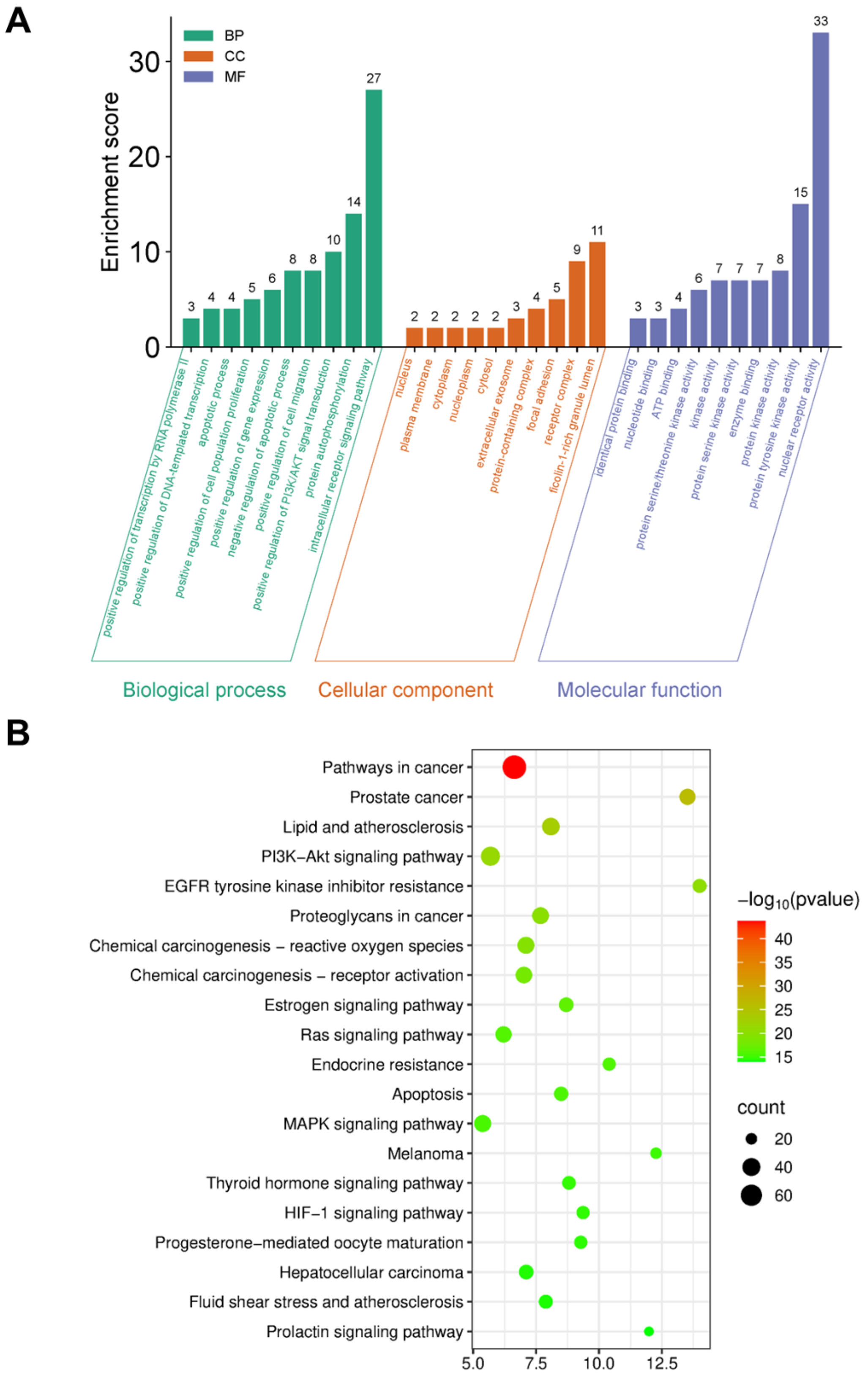

Figure S4. GO enrichment analysis of the candidate targets of chaetomugilin Q in prostate cancer (A). KEGG enrichment analysis of the candidate targets of

chaetomugilin Q in prostate cancer (B).

A

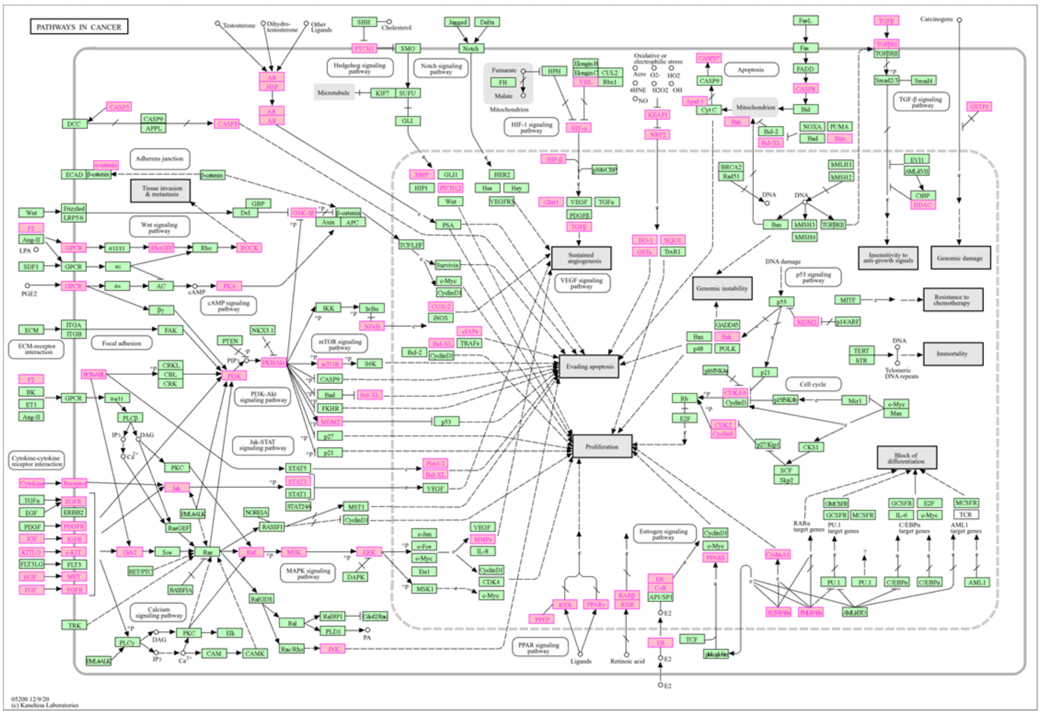

B

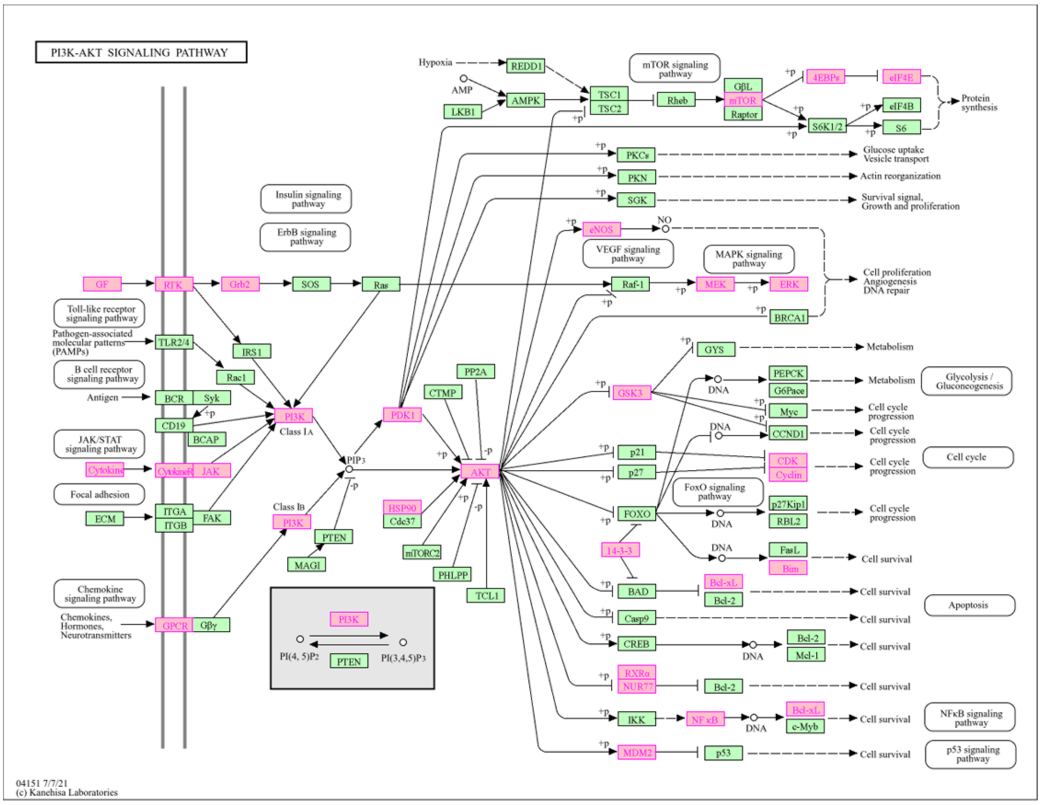

Figure S5. Pathways in cancer (A). PI3K-Akt signaling pathway (B).

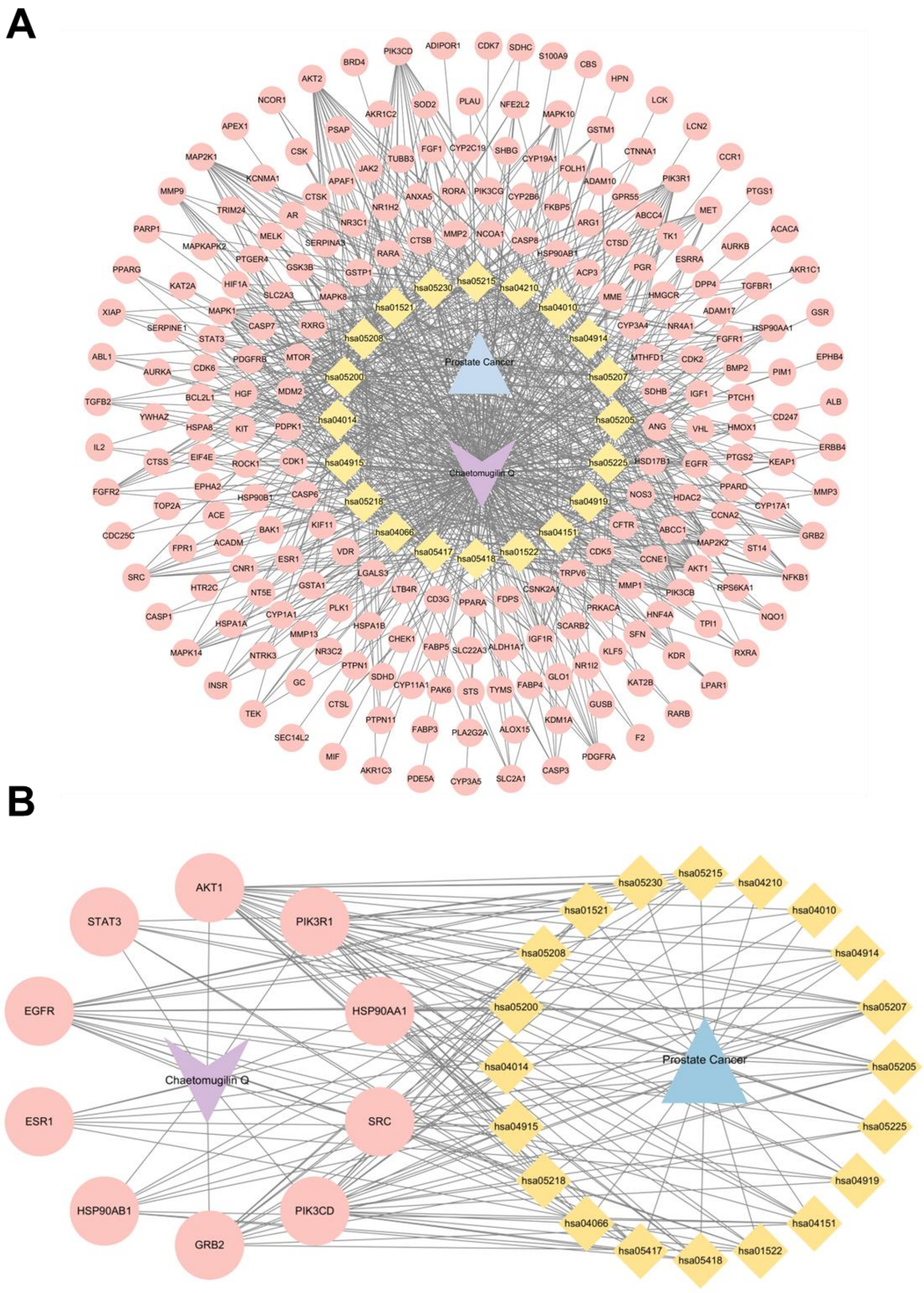

Figure S6. Compound-candidate target–pathway–disease network (A). Compound-candidate core target–pathway–disease network (B).

A

CurPocket ID	Vina score	Cavity volume ($Å^3$)	Center (x, y, z)	Docking size (x, y, z)
⊙C2	-7.8	383	23, 14, 6	23, 23, 23
○C5	-6.2	126	8, 1, 6	23, 23, 23
○C3	-5.5	382	34, 11, 24	23, 23, 23
○C4	-5.2	381	30, -3, 26	23, 23, 23
○C1	-4.8	495	27, 2, 3	23, 23, 23

B

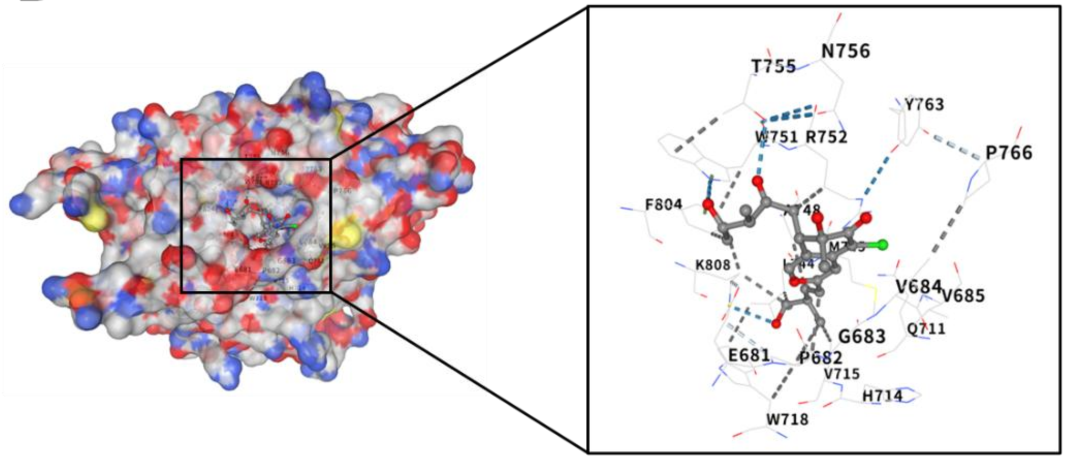

Figure S7. Predicted molecular docking results of chaetomugilin Q with the AR protein (PDB: 1T65) (A). Binding mode of chaetomugilin Q with the AR protein (B).

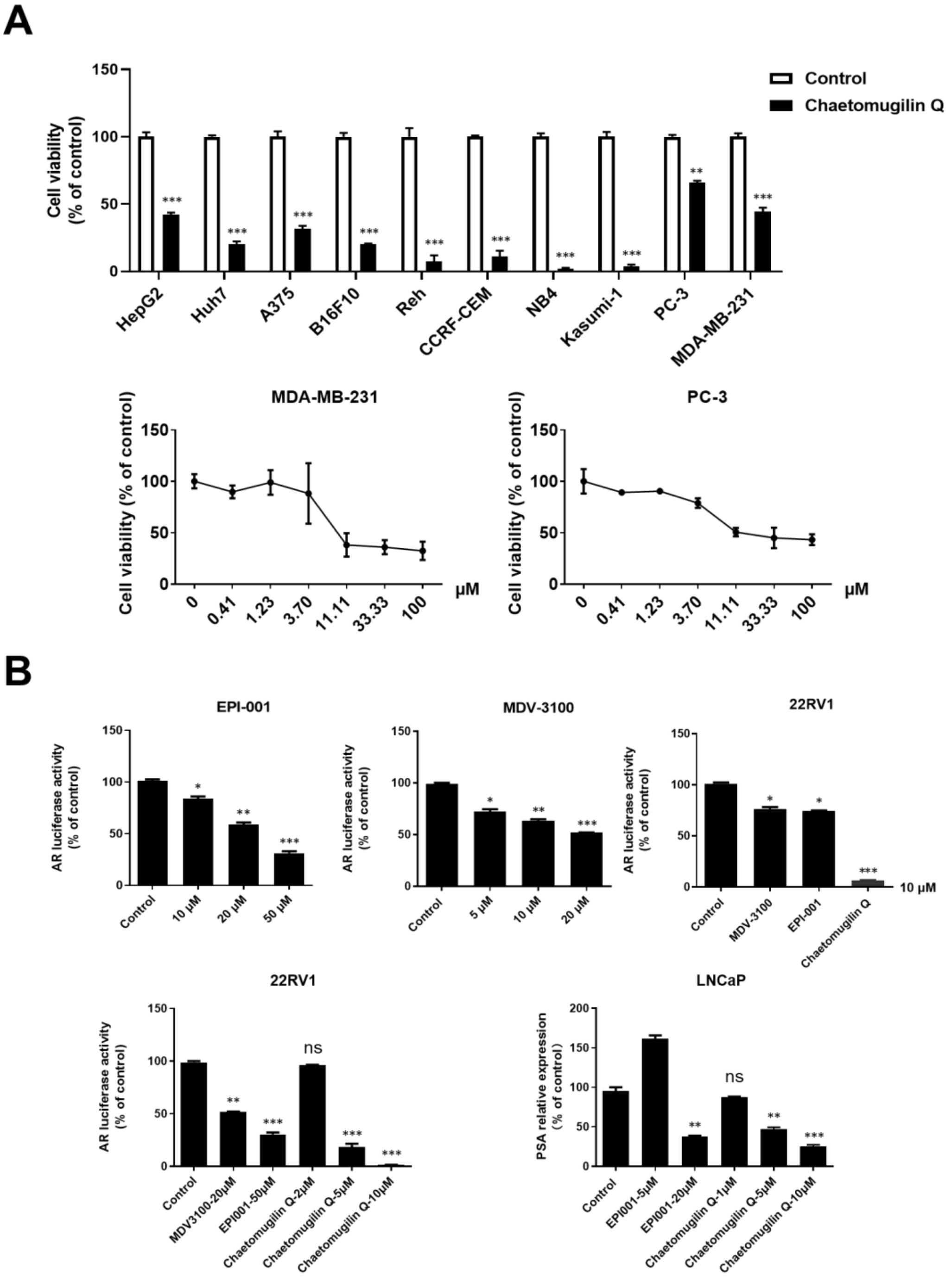

Figure S8. Chaetomugilin Q significantly inhibits the proliferation of various cancer cell lines, including liver cancer (HepG2 and Huh7), melanoma (A375 and B16F10), leukemia (Reh, CCRF-CEM, NB4, and Kasumi-1), prostate cancer (PC-3), and breast cancer (MDA-MB-231) (A). Chaetomugilin Q markedly suppresses AR signaling in AR-positive prostate cancer cells (B). The two AR inhibitors, EPI-001 and MDV3100, were used as positive controls. The data are presented as means ± SD or means ± SEM (n = 3). Statistical significance is indicated as follows: * $p < 0.05$, ** $p < 0.01$, *** $p < 0.001$. ns, not significant.
